# Total Flavonoids of Drynariae Rhizoma Improve Glucocorticoid-Induced Osteoporosis of Rats: UHPLC-MS-Based Qualitative Analysis, Network Pharmacology Strategy and Pharmacodynamic Validation

**DOI:** 10.3389/fendo.2022.920931

**Published:** 2022-06-30

**Authors:** Fangqing Zhang, Qiuyue Li, Jiashuo Wu, Haonan Ruan, Chuanrui Sun, Jia Zhu, Qinghui Song, Xu Wei, Yue Shi, Liguo Zhu

**Affiliations:** ^1^ Wangjing Hospital, China Academy of Chinese Medical Sciences, Beijing, China; ^2^ Institute of Medicinal Plant Development, Chinese Academy of Medical Sciences and Peking Union Medical College, Beijing, China

**Keywords:** total flavonoids of drynariae rhizome, glucocorticoid-induced osteoporosis, network pharmacology, qualitative analysis, PPAR γ

## Abstract

**Background:**

Glucocorticoid-induced osteoporosis (GIOP) is a common form of secondary osteoporosis caused by the protracted or a large dosage of glucocorticoids (GCs). Total flavonoids of Drynariae rhizoma (TFDR) have been widely used in treating postmenopausal osteoporosis (POP). However, their therapeutic effects and potential mechanism against GIOP have not been fully elucidated.

**Methods:**

Ultra-high-performance liquid chromatography coupled with electrospray ionization quadrupole time-of-flight mass spectrometry (UHPLC-ESIQ-TOF-MS) experiments were performed for qualitative analysis. We performed hematoxylin-eosin (HE) staining and microcomputed tomography (micro-CT) analysis to detect the changes in bone microstructure. The changes in biochemical parameters in the serum samples were determined by performing an enzyme-linked immunosorbent assay (ELISA). The prediction results of network pharmacology were verified *via* quantitative real-time polymerase chain reaction (qRT-PCR) to elucidate the potential mechanism of TFDR against GIOP.

**Results:**

A total of 191 ingredients were identified *in vitro* and 48 ingredients *in vivo*. In the *in-vivo* experiment, the levels of the serum total cholesterol (TC), the serum triglyceride (TG), Leptin (LEP), osteocalcin (OC), osteoprotegerin (OPG), bone morphogenetic protein-2 (BMP-2), propeptide of type I procollagen (PINP), tartrate-resistant acid phosphatase (TRACP) and type-I collagen carboxy-terminal peptide (CTX-1) in the TFDR group significantly changed compared with those in the GIOP group. Moreover, the TFDR group showed an improvement in bone mineral density and bone microstructure. Based on the results of network pharmacology analysis, 67 core targets were selected to construct the network and perform PPI analysis as well as biological enrichment analysis. Five of the targets with high “degree value” had differential gene expression between groups using qRT-PCR.

**Conclusion:**

TFDR, which may play a crucial role between adipose metabolism and bone metabolism, may be a novel remedy for the prevention and clinical treatment of GIOP.

**Graphical Abstract:**
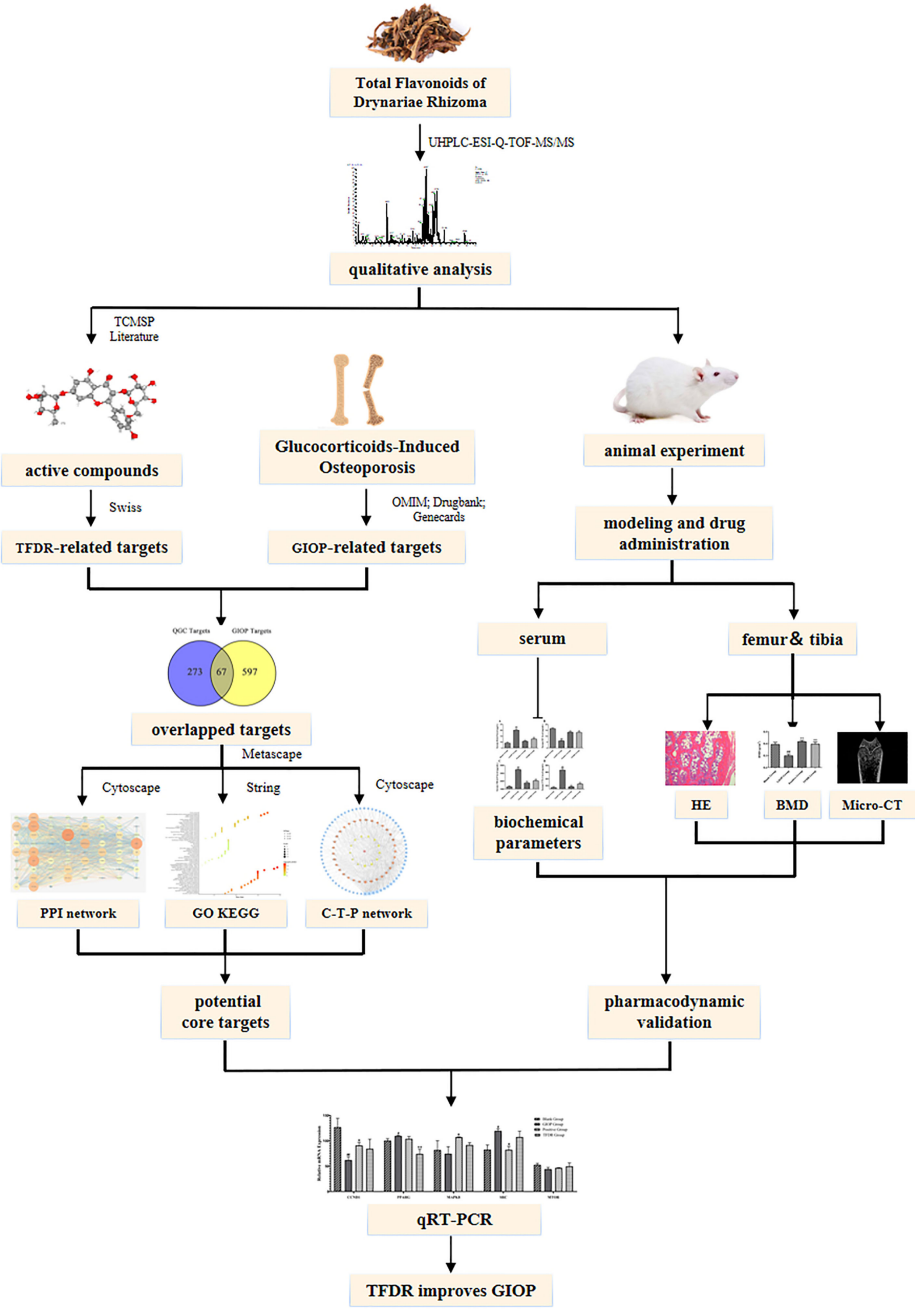


## 1 Introduction

Glucocorticoids (GCs), which are well-known for their unsurpassed efficacy, have been widely used to treat various diseases, especially inflammatory and autoimmune disorders. However, serious adverse outcomes, including osteoporosis, have considerably limited the therapeutic applications of GCs (1). Glucocorticoid-induced osteoporosis (GIOP), a main type of secondary osteoporosis, is caused by the protracted or a large dosage of GCs. Nearly 30-50% of the patients who are administered long-term GC therapy tend to develop GIOP, which is characterized by decreased bone mineral density and deterioration of bone microstructure (2). According to the osteoporosis guidelines (3), patients receiving long-term GC therapy should also be treated for osteoporosis. At present, there are only 4 drugs, including zoledronic acid, alendronate, teriparatide, and risedronate, approved for the treatment of GIOP according to the guidelines of US FDA (United States Food and Drug Administration) (4). While the application and researches of traditional Chinese medicine used to treat GIOP are still scanty. Therefore, developing new drugs or compounds that can enhance the therapeutic efficacy GCs and reduce their side effects holds immense clinical significance.

Traditional Chinese medicine (TCM) has its unique advantages in treating osteoporosis because of the characteristics of multiple ingredients and targets. As a traditional Chinese medicine, Drynariae rhizoma, which is the dried rhizome of *Drynaria fortunei* (Kunze) J. Sm., has a long history of medical use. It is broadly used to treat bone diseases such as osteoporosis, fractures, joint diseases, etc. Many flavonoid compounds have been successfully extracted, identified, and purified from the Drynariae rhizoma extract such as neoeriocitrin, kaempferol, naringin, eriodictyol, luteolin, naringenin, luteolin-7-O-β-D-glucoside, etc. (5). According to previous studies (6–8), total flavonoids of Drynariae rhizoma (TFDR) exhibit promising pharmacological activities, which can possibly improve the bone mineral density (BMD) and change osteocalcin (OC), alkaline phosphatase (ALP) and collagen carboxy-terminal peptide (CTX) levels in the serum of rats. TFDR has been proved to promote osteoclast apoptosis and stimulate osteoblast proliferation (9). Therefore, TFDR is broadly used for osteoporosis treatment. Currently, the researches about the efficacy of TFDR mainly aim to treat postmenopausal osteoporosis (PMOP) and its action mechanism on growth of bone graft and fracture healing (10–12). However, TFDR has not been reported to cure GIOP yet. If TFDR was highly effective for treating this clinical disease which has been recently paid more and more attention to? What were the potential bio-active ingredients of TFDR and its mechanism of action? To solve these questions, we performed our experiment from three parts: potential active ingredients mining, pharmacodynamic validation and mechanism prediction.

For mining potential active ingredients, UHPLC-ESIQ-TOF-MS was used to identify the ingredients both *in-vivo* and *in-vitro*. For mechanism prediction, network pharmacology strategies were applied to select core targets, perform enrichment analysis and construct the “compound-target-pathway” network. For pharmacodynamic validation, a GIOP model in Sprague–Dawley (SD) rats was conducted to perform *in vivo* experiments for assessing the efficacy of TFDR. We performed HE staining, micro-CT and BMD analysis for pharmacodynamic research. The concentration of TC, TG, LEP, OC, PINP, OPG, BMP2, TRACP, CTX-1 in serum was measured using ELISA. Integrating the results of network pharmacology analysis, the core targets were picked out for quantitative real-time polymerase chain reactions (qRT-PCR) to further verify the potential mechanisms. Our study aimed to lay a foundation for the clinical application of TFDR and TFDR was expected to become a potential effective drug to treat GIOP to some extent.

## 2 Materials and Methods

### 2.1 Drug, Animals, Reagents Preparation

Female adult Sprague-Dawley rats (SiPeiFu, Beijing, China); total Flavonoids of Drynariae Rhizoma (TFDR) (Batch number: 200706; Beijing Qihuang Pharmaceutical Co., Ltd, Beijing, China); Dexamethasone (DEX) Sodium-Phosphate Injection (Batch number: 2109205; Sinopharm Ronshyn Pharmaceutical Co., Ltd., Henan, China); Alendronate enteric-coated tablets (Batch number: 266210501; CSPC Pharmaceutical Group Co., Ltd., Shijiazhuang, China); HPLC-grade acetonitrile and formic acid (Honeywell Burdick & Jackson, United States); Ultra-pure water was obtained by using a super-pure water system (Beijing, China); Paraformaldehyde (Macklin Reagent Company, Shanghai, China); Osteocalcin (OC) ELISA kit, Osteoprotegerin (OPG) ELISA kit, Bone morphogenetic protein-2 (BMP-2) ELISA kit, Propeptide of type I procollagen (PINP) ELISA kit, Tartrate-resistant acid phosphatase (TRACP) ELISA kit, Type-I collagen carboxy-terminal peptide (CTX-1) ELISA kit, Leptin (LEP) ELISA kit (Wuhan Myhalic Biotechnological Co., Ltd, Wuhan, China); Total cholesterol (TC) ELISA kit, Triglyceride (TG) ELISA kit (Elabscience, Wuhan, China); Eastep^®^ Super Total RNA Extraction Kit, and Reverse Transcription System (Promega (Beijing) Biotech Co., Ltd, Beijing, China); SYBR^®^ Green Realtime PCR Master Mix (Toyobo Life Science, Shanghai, China).

### 2.2 Ultra-High-Performance Liquid Chromatography Analysis of TFDR

UHPLC-ESIQ-TOF-MS was used for qualitative analysis to identify the compounds of TFDR in this study. All compounds were separated by using a chromatographic instrument equipped with the Waters ACQUITY UPLC HSS T3 C18 column (2.1 × 100 mm, 1.8 μm; Waters Corporation, Milford, MA, USA); U3000 3D-field DAD detector with a wavelength coverage of 200-400 nm; a heated electrospray ionization (HESI) source, etc. The temperature of the analytical column was controlled at 30°C and the injection volume was kept 5 μL each time. The gradient elution conditions were as follows: solvent A (acetonitrile) and solvent B (water with 0.1% v/v formic acid): 0-10 min, 100% B; 10-20 min, 100-70% B; 20-25 min, 70-60% B; 25-30 min, 60-50% B; 30-40 min, 50-30% B; 40-45 min, 30-0% B; 45-60 min, 0% B; 60-60.1 min, 0-100% B; 60.1-70 min, 100% B. The ionization data of each compound were acquired in the positive and negative ion modes at the range of m/z 100-1500. Other operating parameters used for MS analysis were 40 arb for sheath gas flow, 15 arb for auxiliary gas flow rate, 320°C for positive spray voltage, 350°C for Aux gas heater temperature, and 3.2 kV for positive spray voltage. The resolution of MS and MS/MS was set at 70000 and 17500, respectively.

### 2.3 Network Pharmacology

#### 2.3.1 Collection of TFDR-Active Ingredients

The bio-active constituents of TFDR were collected from the pharmacological database of the traditional Chinese medicine system and analyzing platforms (TCMSP, https://old.tcmsp-e.com/tcmsp.php) (13). By using the keywords “*Davallia mariesii* Moore ex Bak.”, 71 herbal compounds were totally identified for the follow-up screening. For these compounds, 18 potential active ingredients were selected with the two key ADME indexes, including oral bio-availability (OB ≥ 30%) and drug similarity (DL ≥ 0.18) (14). When compared with the related literature and collected two-dimensional (2D) structures of each ingredient from the PubChem database (https://pubchem.ncbi.nlm.nih.gov/) (15), 10 flavonoid compounds of TFDR were picked up as the potential candidates.

Furthermore, several ingredients with high pharmacological activities, which can not fulfill the two conditions (OB ≥ 30%, DL ≥ 0.18), were also included according to the results of our previous qualitative analysis or other studies (16).

#### 2.3.2 Compound-Related Targets Prediction

The related targets of active ingredients were predicted *via* the Swiss Target Prediction platform (http://www.swisstargetprediction.ch/index.php, updated at 20 May 2019) (17), which serves as an ideal database containing over 3000 proteins and 370,000 active ingredients. Two-dimensional (2D) structures of each active ingredient collected from the PubChem database were then imported into the Swiss platform with *Homo sapiens* as the restriction. Then, the platform identified the potential probable targets according to the structure’s similarity. After deleting the duplication and low probability targets, 273 targets of TFDR were finally selected. To increase the prediction accuracy and ensure clarity of the results, the predictive analysis was performed only on the Swiss Target Prediction platform.

#### 2.3.3 GIOP-Related Targets Prediction

Human glucocorticoids-induced osteoporosis targets were annotated and predicted *via* three pathways: the Online Mendelian Inheritance in Man (OMIM, https://omim.org/, updated at 10 November 2021) (18); GeneCards (https://www.genecards.org/, updated at 10 November 2021) (19); Drugbank (https://go.drugbank.com/, updated at 10 November 2021) (20). The search term “glucocorticoids-induced osteoporosis” was applied to collect the target information including the name and gene ID, and the search results were normalized using the UniProt database (https://www.uniprot.org/, updated at 19 October 2021) (21). After deleting duplicates, the detailed information of 530 GIOP-related targets was integrated to construct a disease target database for subsequent analyses.

#### 2.3.4 Network Construction and Enrichment Analyses

The Cytoscape 3.7.2 (https://cytoscape.org/) (22) was used to construct the network of the bio-active compounds and their related targets. The degree value, which was determined using the Cytoscape Network Analyzer, represents the closeness between the nodes in the network. The scale of nodes is proportional to the closeness; in other words, the importance of the node increases with its increasing size.

The pathways that can indicate the potential functional roles of targets were evaluated *via* the Kyoto Encyclopedia of Genes and Genomes (KEGG) pathway enrichment analysis and the Gene Ontology (GO) pathway enrichment analysis with the application of Metascape (https://metascape.org/) (23). The overlapped targets were imported into the platform, where count ≥ 3, p < 0.01, enrichment factor > 1.5 served as the screening condition. The GO analyses, including the biological process (BP), cellular component (CC), and molecular function (MF) terms, can help to effectively reveal the potential biological molecular mechanisms. To make the enrichment results of GO and KEGG analyses more intuitive, they were visualized as a bubble chart, respectively. Furthermore, the intersection of the compound-related targets and GIOP-related targets was selected to create the compound-target-pathway interaction network.

#### 2.3.5 Protein-Protein Interaction Analysis

Based on the STRING 11.0 (https://string-db.org/) (24), a protein-protein interaction (PPI) analysis served as a structural model to describe the potential. The relationship between every crucial target selected earlier. The files with the suffix “TSV” were collected by entering the gene names, setting the parameter organism as Homo sapiens, and keeping other basic settings as default. Using the Cytoscape 3.7.2 software, the degree and combined scores were assessed, and the PPI network of the common targets was conducted. Incidentally, all the targets, including the abovementioned gene symbols and protein descriptions have been confirmed and normalized using the Uniprot database. Then a total of top 10 targets with high degree value were chose to validate according to the “degree value” of network pharmacology, which were then introduced in more details in 2.4.6.

### 2.4 Pharmacodynamic Validation

#### 2.4.1 Animals Experiment

Female Sprague-Dawley (SD) rats (200-220 g) used to verify the efficacy of TFDR were purchased from Sibeifu Beijing Biotechnology Co., Ltd. (Beijing, China) (SCXK 2019-0010). After the 1-week adaptive feeding in an SPF-class environment, all rats were randomly classified into four groups (n = 6): the blank group, the GIOP group, the positive group, and the TFDR-treated group. Three groups (except the blank group) were administered with dexamethasone (2.5 mg/kg, dissolved in 20 mL saline) *via* intramuscular injection, while the blank group was administered the same amount of physiological saline. After 1-week of modeling, TFDR were administered to the TFDR-treated group *via* intragastric administration once a day (63 mg/kg, dissolved in 250 mL distilled water), and the positive group received alendronate (1.517 mg/kg, dissolved in 500 mL distilled water). Meanwhile, the blank and GIOP groups received the same amount of saline daily. Each rat was weighed at a weekly interval with the drug’s dose adjusted according to the weight gain. The rats were anesthetized with an intraperitoneal injection of Ulatan. The serum and the femur of each rat were finally collected after the 9-week modeling. All the samples were stored in the -80°C fridge for further analysis. All the operations during the experiment were satisfied with the ARRIVE guidelines (25). All the animal experiments were approved by the Experimental Animal Center of IMPLAD (SLXD 20211029231), and the experimental procedures were performed in strict accordance with Legislation Regarding the Use and Care of Laboratory Animals of China. The equivalent dose for rats was obtained using the formula as follows: Animal equivalent dose (mg/kg) = Human dose (mg/kg) × Human Km ÷ Animal Km.

#### 2.4.2 Ingredients Ingested Into Blood by UHPLC Analysis

The serum samples of each group were thawed at 4°C and acetonitrile (3:1) was added to precipitate the proteins. After centrifugation, the supernatants were collected for further analyses. The serum samples were analyzed *via* UHPLC-ESIQ-TOF-MS under the conditions mentioned in Section 2.2.

#### 2.4.3 Biochemical Parameters

The blood samples were collected and allowed to stand for 30 min. After centrifuging at 4000 rpm for 10 min at 4°C, the upper serum samples were obtained and stored in the -80°C fridge for subsequent analyses. The concentrations of serum bone-formation markers (i.e., OC, OPG, PINP and BMP-2) and bone resorption markers (i.e., TRACP and CTX-1) were determined by using detection kits listed under the Materials section. Besides, the concentration of TC, TG, and LEP was also measured by ELISA kit.

#### 2.4.4 Hematoxylin-Eosin Staining

The femur samples from all rats were fixed in 4% paraformaldehyde solution for 72 h. At room temperature, the fixative femurs were decalcified with 20% EDTA (pH 7.4) for 4 weeks before paraffin embedding. Then, the paraffin-embedded sections were cut into 5-μm thickness slices that were then stained with hematoxylin and eosin according to the HE staining method and observed by light microscopy.

#### 2.4.5 Bone Mineral Density and Micro-Computed Tomography Analysis

A high-resolution X-ray Microtomograph (micro-CT; Skyscan1276, Bruker, Belgium) was utilized to scan the right femur of each rat. The bone mineral density (BMD, g/cm^3^) of the same femur was also measured by this machine. Then, the 3-mm area in length from 1.5-mm below the growth plate of the femoral medial was set as the region of interest (ROI) for three-dimensional reconstruction. The obtained three-dimensional images were analyzed by the N-Recon software and followed by CT-AN software for analyses. The percentage of bone volume per tissue volume (BV/TV, %), trabecular thickness (Tb. Th), trabecular separation (Tb. Sp), and trabecular number (Tb. N) were obtained for subsequent analyses. The bone mineral density (BMD, g/cm^3^) of the same femur was also measured by this machine.

#### 2.4.6 Quantitative Real-Time Polymerase Chain Reaction (qRT-PCR)

We chose a total of top 10 targets with high degree value to validate according to the”degree value”of network pharmacology. Based on the guideline from MIQE standard (http://www.rdml.org/) (26), total RNAs of the femur were extracted using the total RNA extraction kit (Promega, China) on an ice bath. To ensure purity and integrity, the RNA extracts were detected by the Nanodrop 2000C spectrophotometer and the Bio-RAD electrophoresis apparatus. Then, the RNAs were reverse-transcribed to cDNA with the primers (Sangon Biotech, China) and reverse transcriptase (Promega, China). The sequences of the reverse transcription primers were shown in **Table 1**. The Real-time PCR Master Mix (Toyobo, China) that aimed to create the PCR reaction system was used and subjected to the Realtime-PCR on the PCR apparatus (Bio-RAD, United States) in a total reaction volume of 20 μL with the following condition: 95°C for 15 s, 40 cycles of 60°C for 15 s, and 72°C for 45 s. The gene expression was calculated according to the 2^−ΔΔCT^ method.

**Table 1:** PCR primer design.

Gene name	Forward primer (5’-3’)	Reverse primer (3’-5’)
CCND1	GAGGCGGATGAGAACAAGCAGATC	GGAGGGTGGGTTGGAAATGAACTTC
PPARγ	CGCCAAGGTGCTCCAGAAGATG	AGGGTGAAGGCTCATATCTGTCTCC
MAPK8	CACAGTGAGCAGAGCAGGCATAG	TTGTCAGGAGCAGCACCATTCTTAC
SRC	GTCGCCTCCCTTCATCCTCTCTC	TACCAGCCTCAACCTGTCCTTCC
MTOR	CCATCTCGGCAACTTGACCATCC	AAGTGCTGCATGTGCTGGAAGG

### 2.5 Statistical Analyses

SPSS 23.0 software was used to evaluate the data and the results were presented as mean ± SEM. The comparisons between the groups were conducted by one-way analysis of variance. p < 0.05 was considered to be statistically significant, and p < 0.01 was considered to be statistically highly significant.

## 3 Results

### 3.1 Chemical Constituents of TFDR

**Figure 1:**
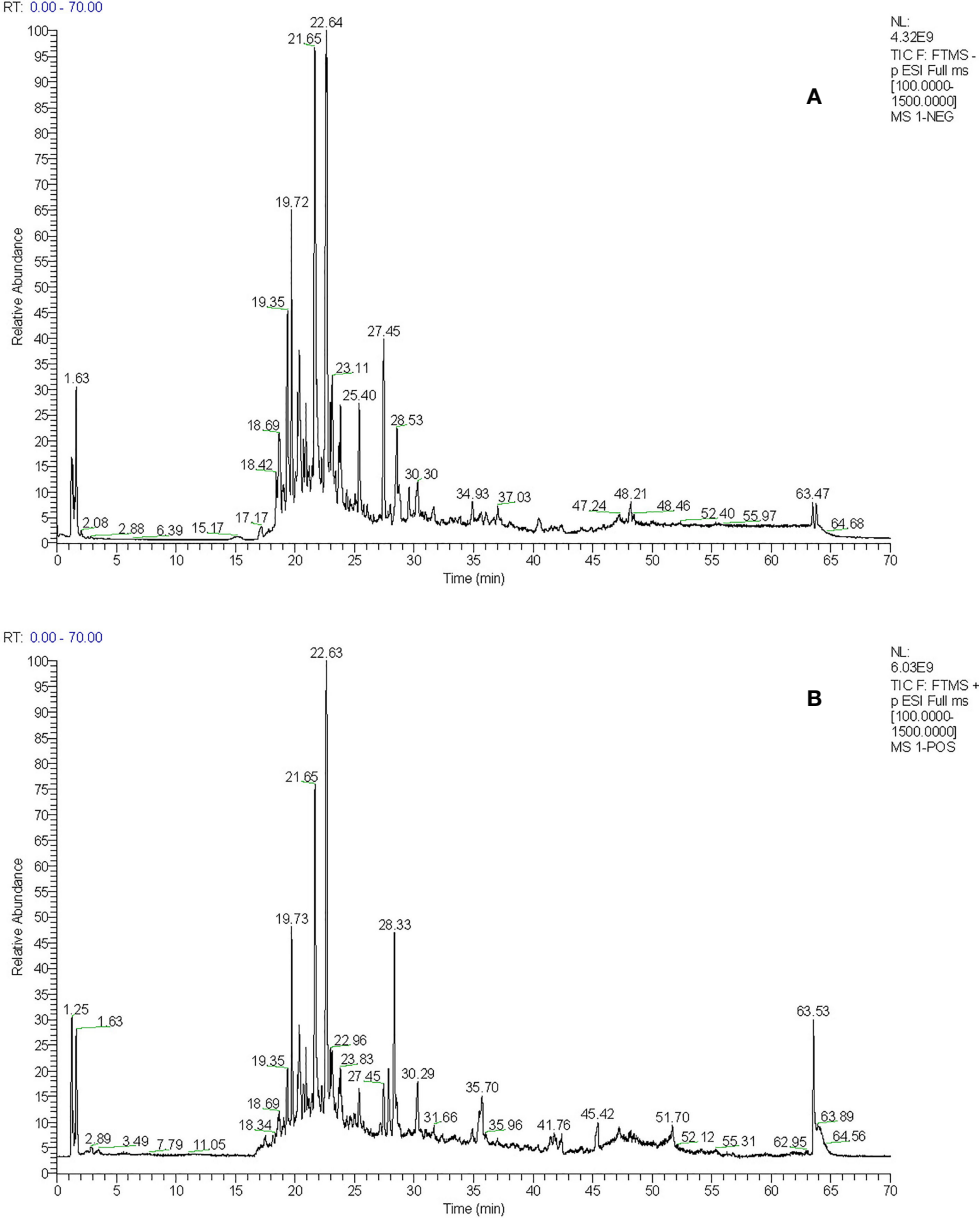
**(A)** The total ion chromatogram of TFDR in negative ion mode **(B)** The total ion chromatogram of TFDR in positive ion mode.

The total ion flow chromatogram of TFDR was obtained by performing UHPLC-ESI-Q-TOF-MS analysis (**Figure 1**). A total of 191 constituents, including naringin, naringenin, kaempferitrin, kaempferol, luteolin, eriodictyol, cianidanol, etc. (**Supplementary Table S1**), were then identified. The constituents were arranged in order according to retention time and additional information (molecular formula, molecular mass, identity, error, ion mode) was listed.

### 3.2 Network Pharmacology

#### 3.2.1 Bio-Active Compounds of TFDR and Target Identification

**Figure 2:**
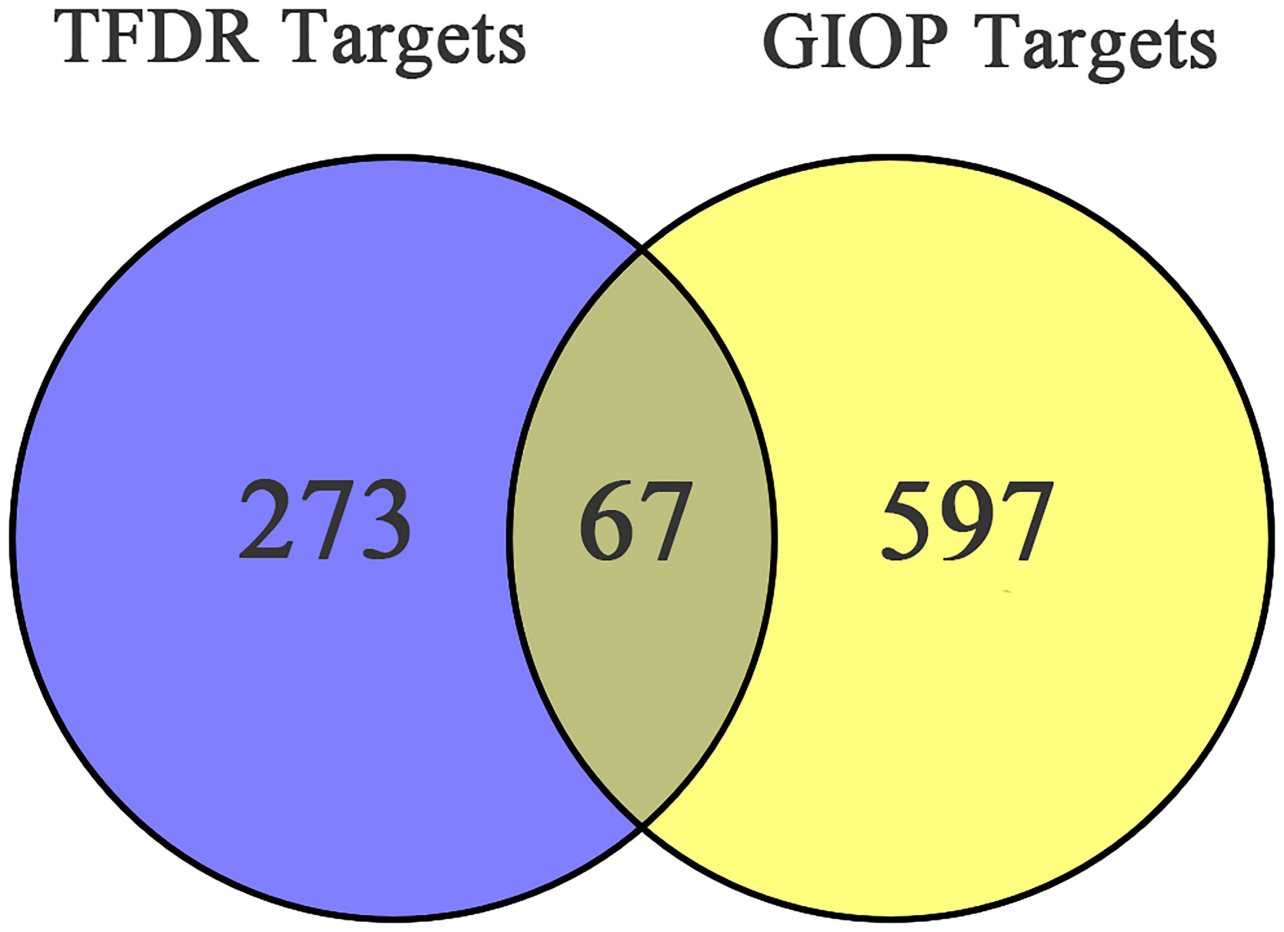
The Venn diagram of targets in TFDR and GIOP.

After screening, 13 bioactive compounds (**Supplementary Table S3**) were selected from the TCMSP database, literature search, and qualitative analysis results (3 compounds from the TCMSP database, 7 from qualitative analysis results and 3 from literature search). Based on the Swiss Target Prediction platform, 273 compound-related targets were identified. A total of 597 GIOP-related targets were identified by searching the OMIM, GeneCards, and Drugbank databases. The Venn diagram (**Figure 2**) showed that 67 targets (**Table 2**) were at the intersection of compound targets and disease targets, which were also predicted as the potential targets of TFDR for alleviating GIOP.

**Table 2:** The information of 67 overlapped targets.

Target name	Full name of target
ABCB1	ATP-dependent translocase ABCB1
ABCC1	Multidrug resistance-associated protein 1
ABCG2	Broad substrate specificity ATP-binding cassette transporter ABCG2
AHR	Aryl hydrocarbon receptor
AKR1C1	Aldo-keto reductase family 1 member C1
AKR1C2	Aldo-keto reductase family 1 member C2
AKT1	RAC-alpha serine/threonine-protein kinase
APP	Amyloid-beta precursor protein
AR	Androgen receptor
BCL2	Apoptosis regulator Bcl-2
BCL2L1	Bcl-2-like protein 1
BRAF	Serine/threonine-protein kinase B-raf
CCND1	G1/S-specific cyclin-D1
CCNE1	G1/S-specific cyclin-E1
CDK2	Cyclin-dependent kinase 2
CDK5	Cyclin-dependent-like kinase 5
CFTR	Cystic fibrosis transmembrane conductance regulator
CSF1R	Macrophage colony-stimulating factor 1 receptor
CTSB	Cathepsin B
CTSK	Cathepsin K
CYP19A1	Aromatase
CYP3A4	Cytochrome P450 3A4
DYRK1A	Dual specificity tyrosine-phosphorylation-regulated kinase 1A
ERBB2	Receptor tyrosine-protein kinase erbB-2
ERN1	Serine/threonine-protein kinase/endoribonuclease IRE1
ESR1	Estrogen receptor
ESR2	Estrogen receptor beta
GSK3B	Glycogen synthase kinase-3 beta
HNF4A	Hepatocyte nuclear factor 4-alpha
HSP90AA1	Heat shock protein HSP 90-alpha
IGFBP3	Insulin-like growth factor-binding protein 3
IL2	Interleukin-2
INSR	Insulin receptor
LCK	Tyrosine-protein kinase Lck
MAPK8	Mitogen-activated protein kinase 8
MAPT	Microtubule-associated protein tau
MDM2	E3 ubiquitin-protein ligase Mdm2
MMP1	Interstitial collagenase
MMP7	Matrilysin
MMP9	Matrix metalloproteinase-9
MTOR	Serine/threonine-protein kinase mTOR
NCSTN	Nicastrin
NOS2	Nitric oxide synthase, inducible
NR1H4	Bile acid receptor
PARP1	Poly [ADP-ribose] polymerase 1
PGF	Placenta growth factor
PIK3CA	Phosphatidylinositol 4,5-bisphosphate 3-kinase catalytic subunit alpha isoform
PIK3CB	Phosphatidylinositol 4,5-bisphosphate 3-kinase catalytic subunit beta isoform
PIK3CG	Phosphatidylinositol 4,5-bisphosphate 3-kinase catalytic subunit gamma isoform
PIK3R1	Phosphatidylinositol 3-kinase regulatory subunit alpha
PPARγ	Peroxisome proliferator-activated receptor gamma
PRKCD	Protein kinase C delta type
PSEN1	Presenilin-1
PTGER2	Prostaglandin E2 receptor EP2 subtype
PTGS1	Prostaglandin G/H synthase 1
PTGS2	Prostaglandin G/H synthase 2
PTK2	Focal adhesion kinase 1
PTPRS	Receptor-type tyrosine-protein phosphatase S
RPS6KA3	Ribosomal protein S6 kinase alpha-3
SERPINE1	Plasminogen activator inhibitor 1
SLC5A1	Sodium/glucose cotransporter 1
SRC	Proto-oncogene tyrosine-protein kinase Src
TERT	Telomerase reverse transcriptase
TLR9	Toll-like receptor 9
TNF	Tumor necrosis factor
TXK	Tyrosine-protein kinase TXK
VEGFA	Vascular endothelial growth factor A

#### 3.2.2 Network Construction and Enrichment Analysis

**Figure 3:**
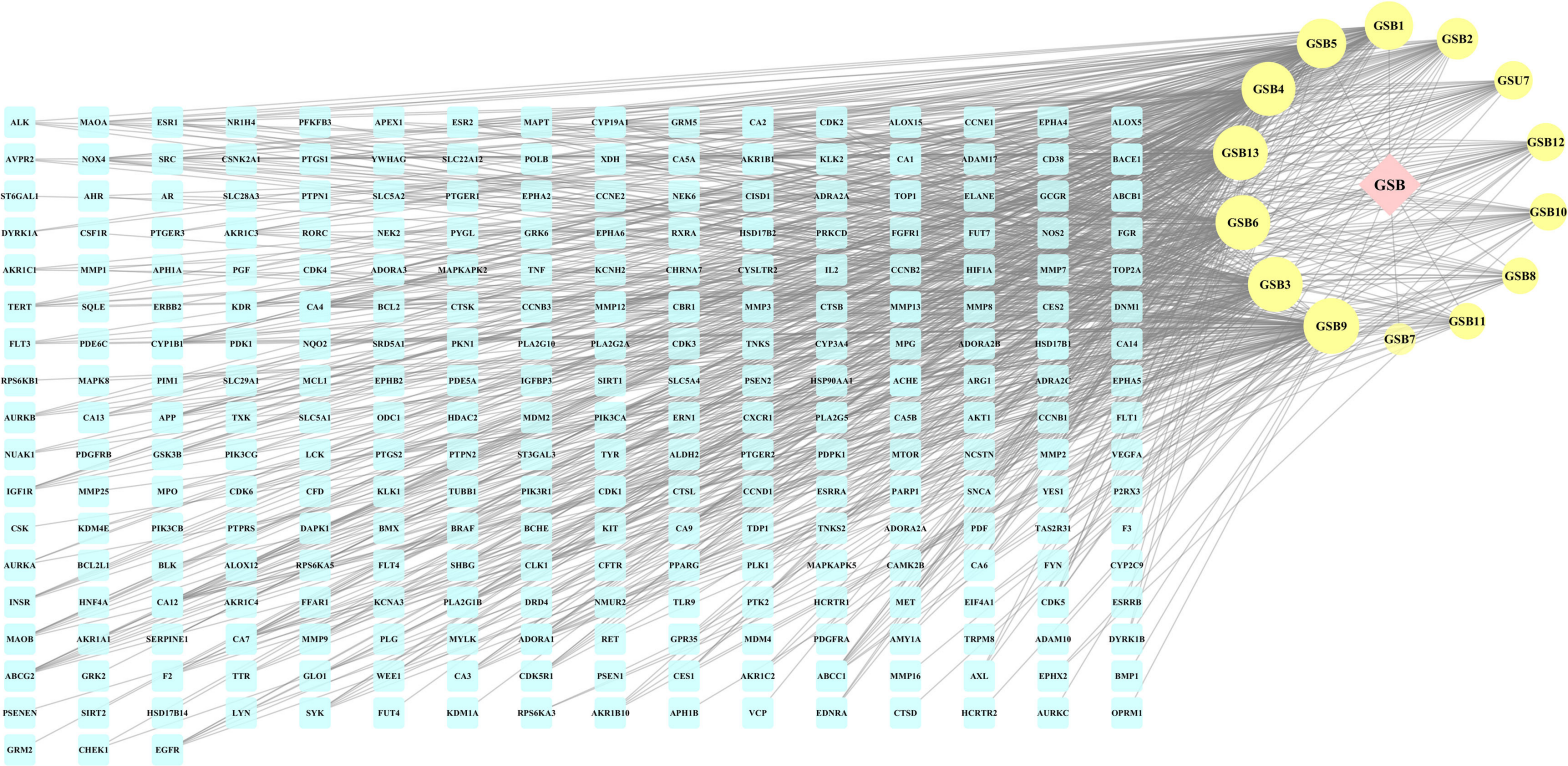
The herb-compounds-targets network of 287 nodes. The pink node represents TFDR, the light-yellow nodes represent its compounds and the light blue nodes represent the gene symbols. The size of node is proportional to the degree value and the edges represent interactions between the nodes.

**Figure 4:**
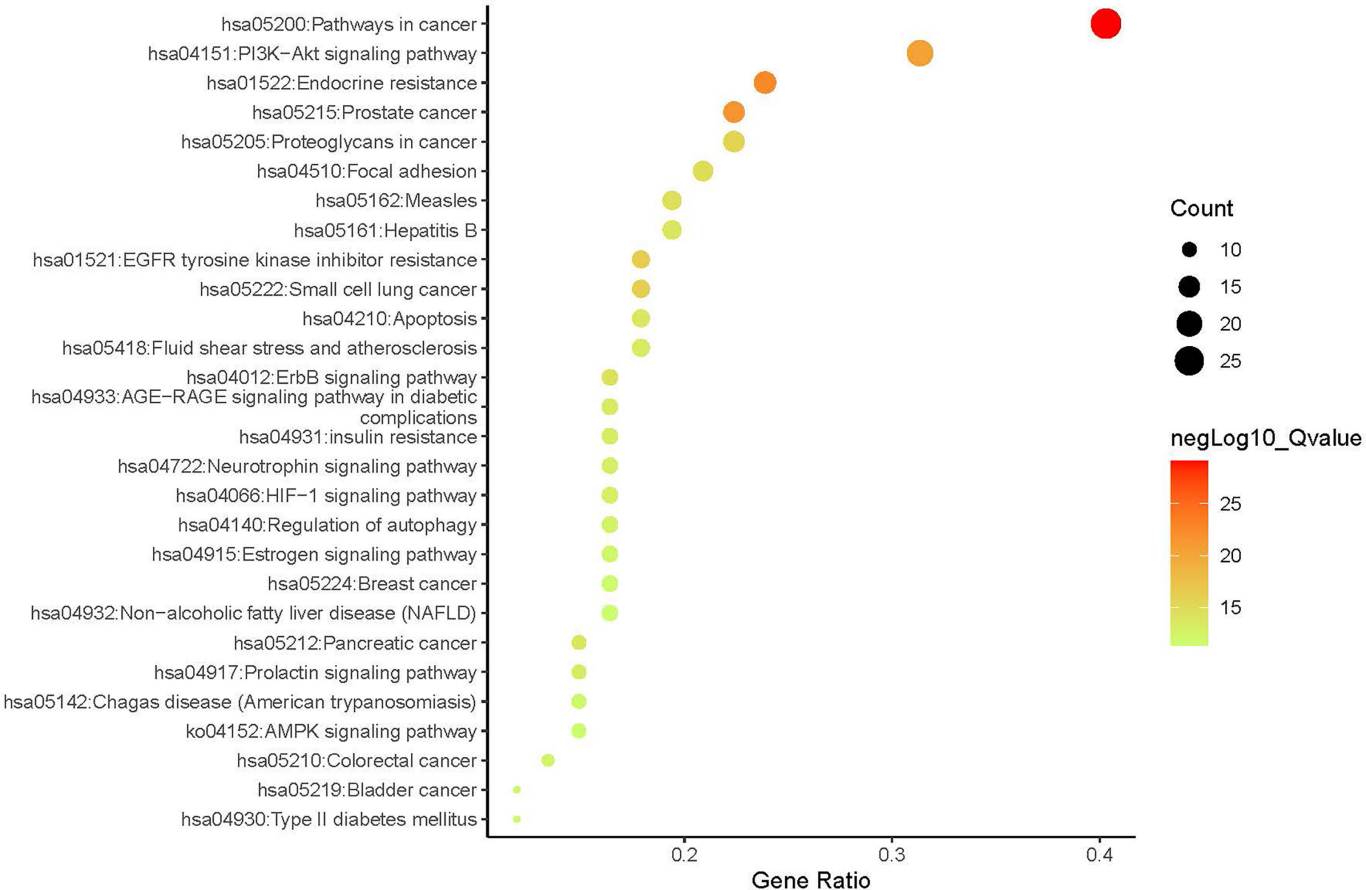
The Enrichment plot of KEGG pathway analysis. The size and color of nodes represent the count and –log10(q) values of pathways.

**Figure 5:**
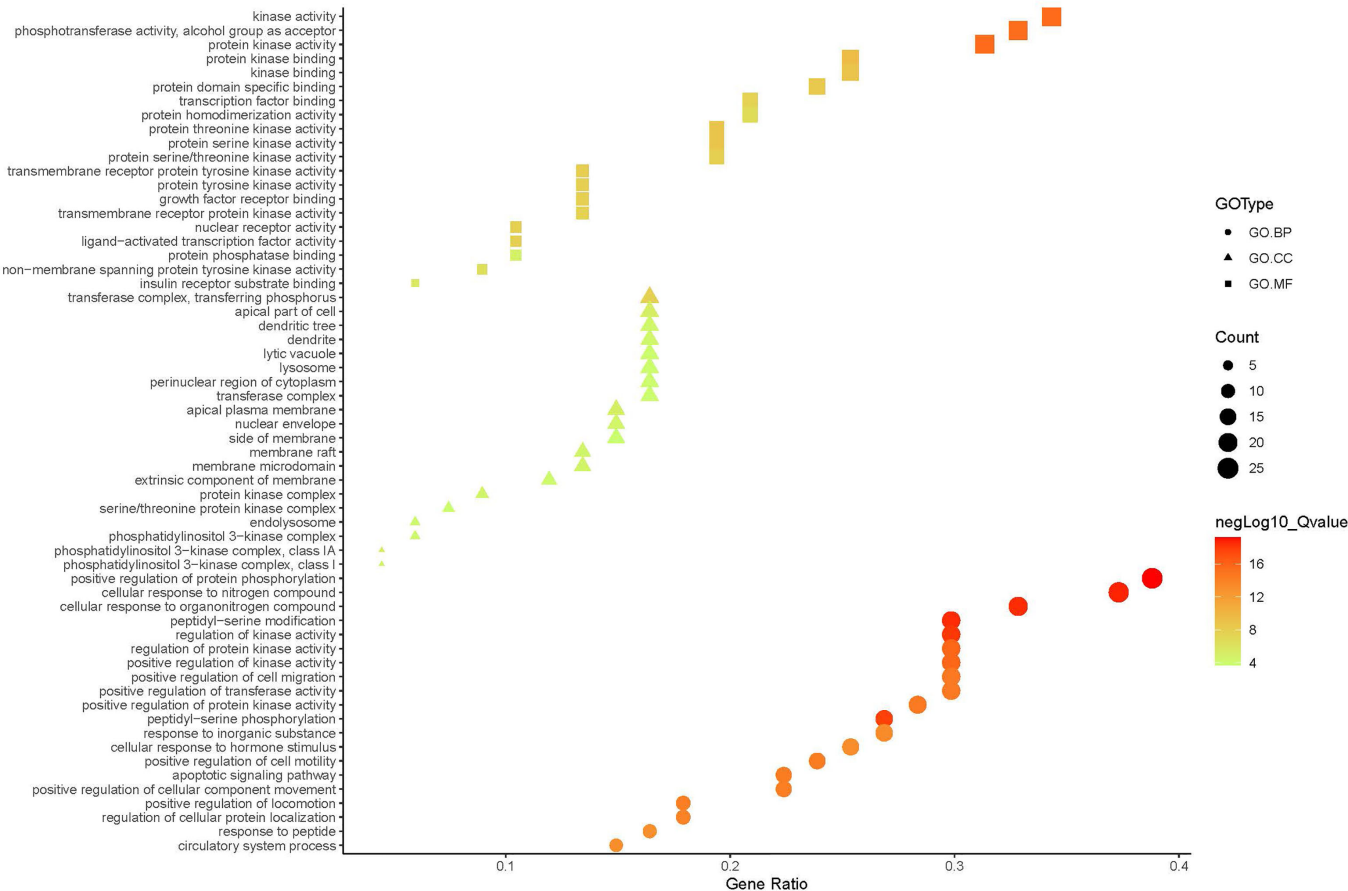
The GO.MF, GO.BP and GO.CC enrichment analysis plot of TFDR. The size and color of nodes represent the count and –log10(q) values of molecular functions, biological processes, and cellular components items.

**Figure 6:**
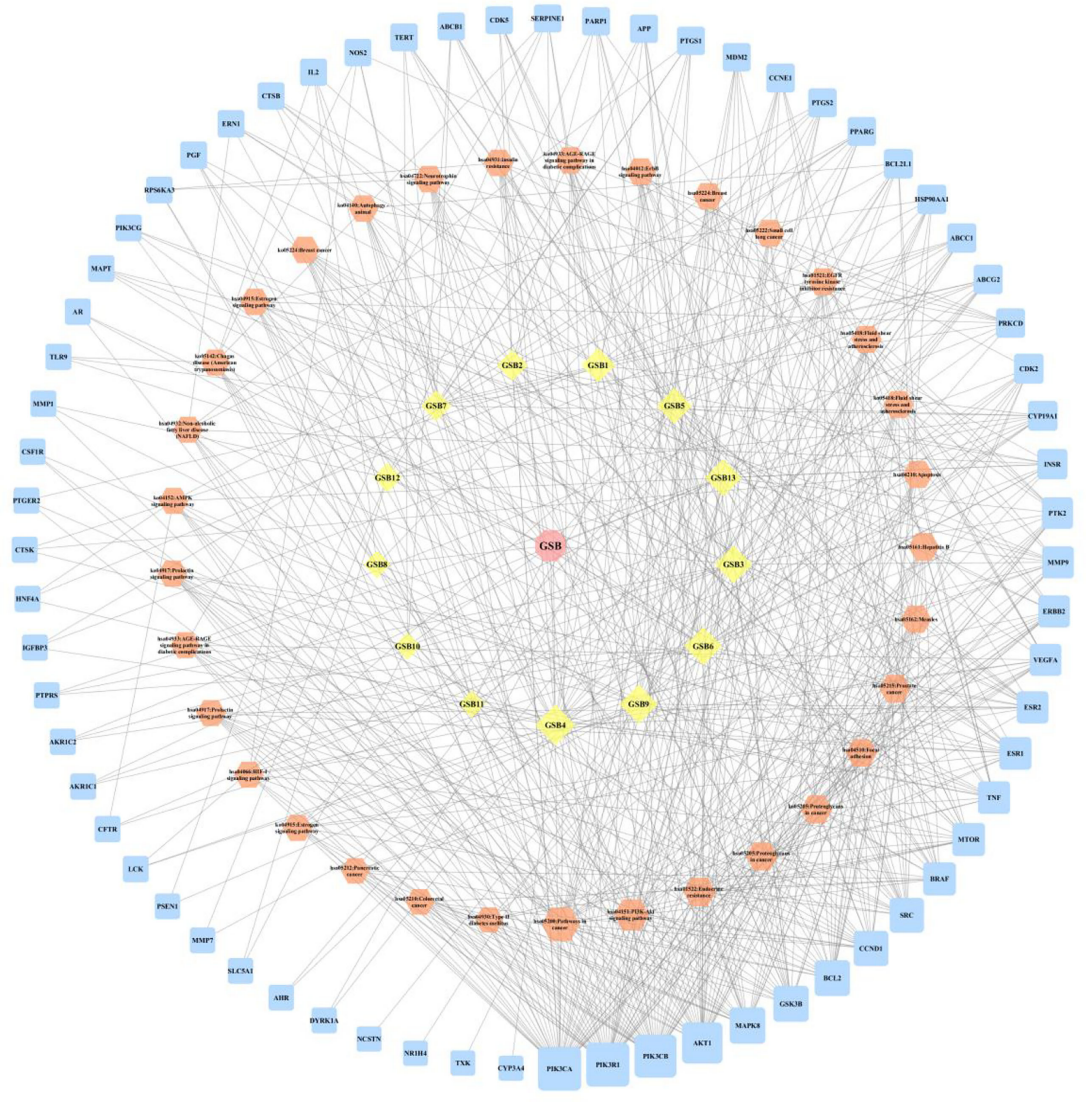
The herb-compounds-targets-pathways network of TFDR. The light pink nodes represent TFDR, the light-yellow nodes represent its 13 compounds, the light orange nodes represent pathways and the light blue nodes represent the gene symbols. The size of node is proportional to the degree value and the edges represent interactions between the nodes.

A compound-target network (**Figure 3**) including 13 nodes for bioactive compounds and 273 nodes for compound-related targets was constructed. The Metascape platform was used to perform GO and KEGG enrichment analyses. The top 20 results with high q-values were selected to be the most relevant pathways (**Figures 4**, **5**). The pathways contributing to cancer development and the PI3K/AKT signaling pathway showed a high correlation in both GO and KEGG analyses results, implying that these pathways might be closely linked to the pharmacological activities of TFDR involved in improving GIOP. Based on the above results, a herb-compound-target-pathway network was then established (**Figure 6**). Kaempferol, luteolin, and naringenin with a high degree value might be the key compounds in TFDR that may act against GIOP and the PI3K/AKT signaling pathway might be involved in the potential mechanism underlying the action of TFDR against GIOP.

#### 3.2.3 PPI Network Analysis

**Figure 7:**
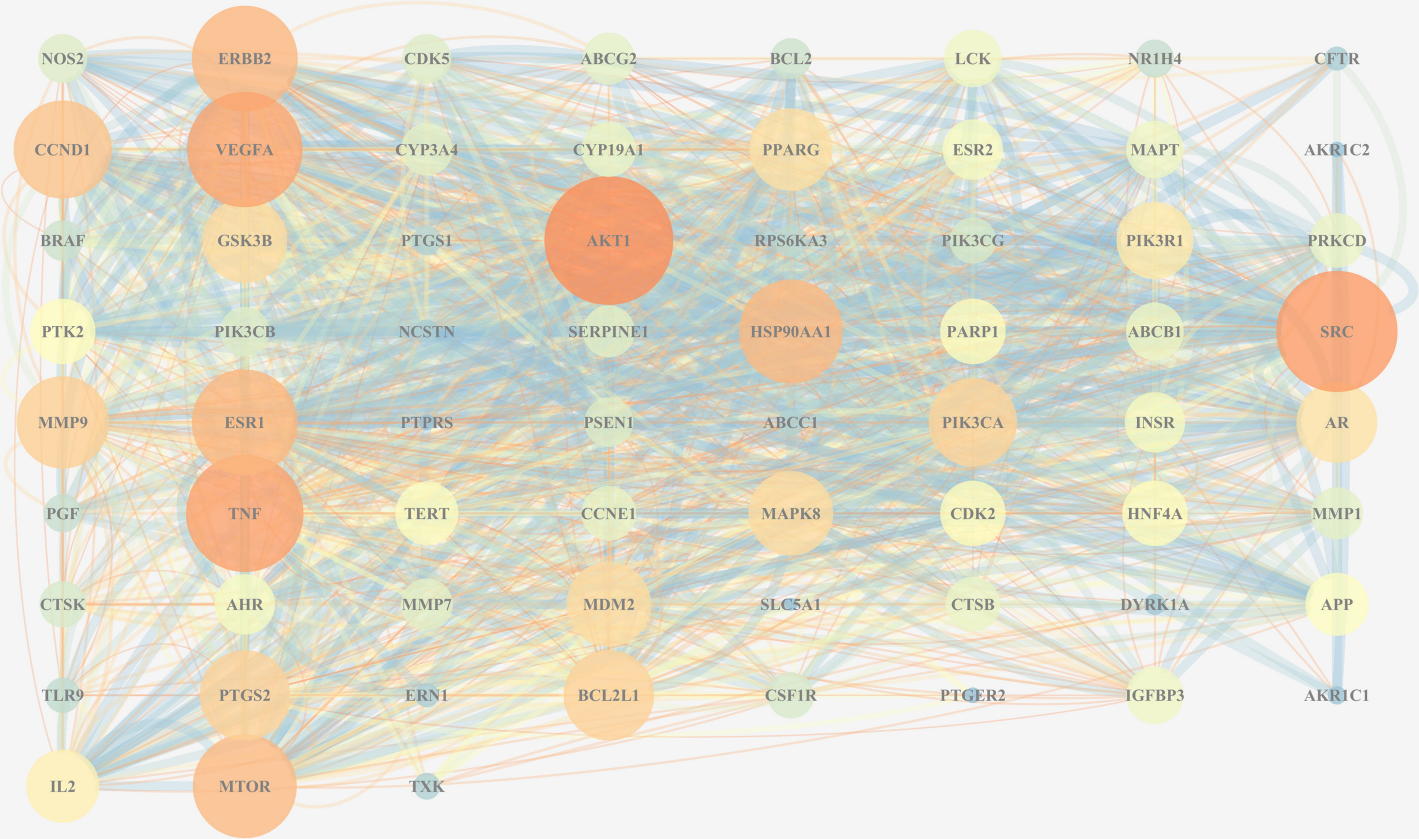
The PPI network of all overlapped targets. The size of nodes represents their degree values, and the width of edges represents interactions between the nodes. The color represents their degree values and combined scores, which changes from blue to orange represent values from low to high.

To further explore the protein-interaction relationship among the 67 targets, String 11.0 was used to construct a PPI network. The Cytoscape 3.7.2 software was used to visualize the analysis results (**Figure 7**). As is shown in the network diagram in **Figure 7**, AKT1, SRC, TNF, VEGFA, and ESR1 played a significant role because of a high degree value, suggesting that these targets may be the potential candidates in TFDR-mediated GIOP treatment.

### 3.3 *In Vivo* Experiments

#### 3.3.1 Identification of Chemical Constituents Ingested Into Blood

A total of 48 constituents found in the serum samples were identified (**Supplementary Table S2**) by comparing the data from mzCloud and mzVault databases. The constituents luteolin-7-glucuronide, naringenin, naringenin chalcone and eriodictyol, which have been reported earlier to exhibit considerable pharmacological activity, were also detected in the serum samples.

#### 3.3.2 Effects of TFDR on Serum Biochemical Parameters (Bone Formation and Resorption Markers)

**Figure 8:**
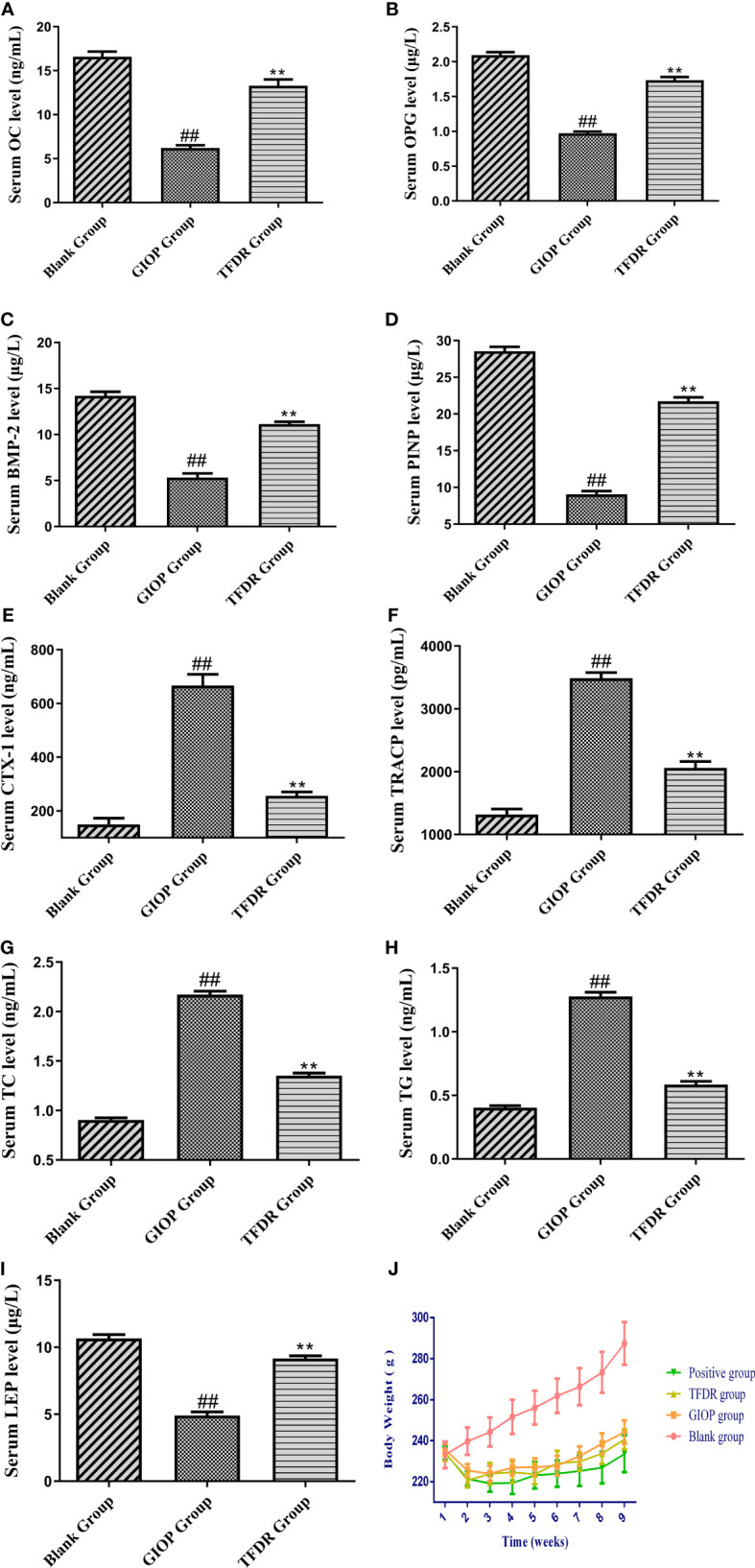
The serum biochemical parameters including OC **(A)**, OPG **(B)**, BMP-2 **(C)**, PINP **(D)**, CTX-1 **(E)**, TRACP **(F)**, TC **(G)**, TG **(H)**, LEP **(I)** and the changes of body weight **(J)**. Data are expressed as mean ± SEM. *p < 0.05 and **p < 0.01 vs. GIOP group rats; ^#^p < 0.05 and ^##^p < 0.01 vs. blank group rats (n = 6).

After modeling, the concentration of OC, OPG, BMP-2 and PINP in the serum samples of the GIOP group was significantly lower than those in the blank group, whereas the concentration significantly increased after administrating TFDR (**Figures 8A–D**), indicating that TFDR could reverse DEX-mediated loss of bone. The expression of bone resorption markers (i.e., TRACP and CTX-1) was significantly upregulated in GIOP rats, whereas that of TRACP and CTX-1 in the TFDR group returned to normal (**Figures 8E, F**). Besides, the levels of serum TC, TG in TFDR group showed a significant decline compared with them in GIOP group, while the level of serum LEP in the treatment group showed a raising trend (**Figure 8G–I**), indicating TFDR had an effect on lipid metabolism. Besides, the weight of rats in GIOP group and TFDR group was significantly decreased compared with those in the blank group (**Figure 8J**).

#### 3.3.3 Effects of TFDR on Bone Mineral Density, Bone Microstructure, and Bone Histological Changes

The mean femoral BMD value in the GIOP group was 0.300 ± 0.023, which showed a significant decrease compared with that in the blank group (0.396 ± 0.032; p < 0.01). After treatment with TFDR and the positive drug, the BMD value increased (p < 0.01), indicating that TFDR can improve bone density (**Figure 10A**).

**Figure 9:**
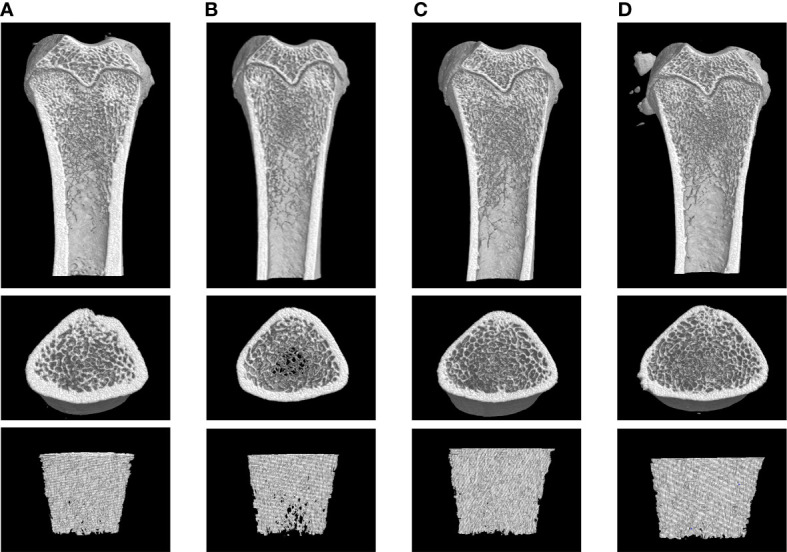
Representative 3D reconstructions of micro-CT analysis of the femur from Blank **(A)**, GIOP **(B)**, Positive **(C)** and TFDR **(D)** group (n = 6).

**Figure 10:**
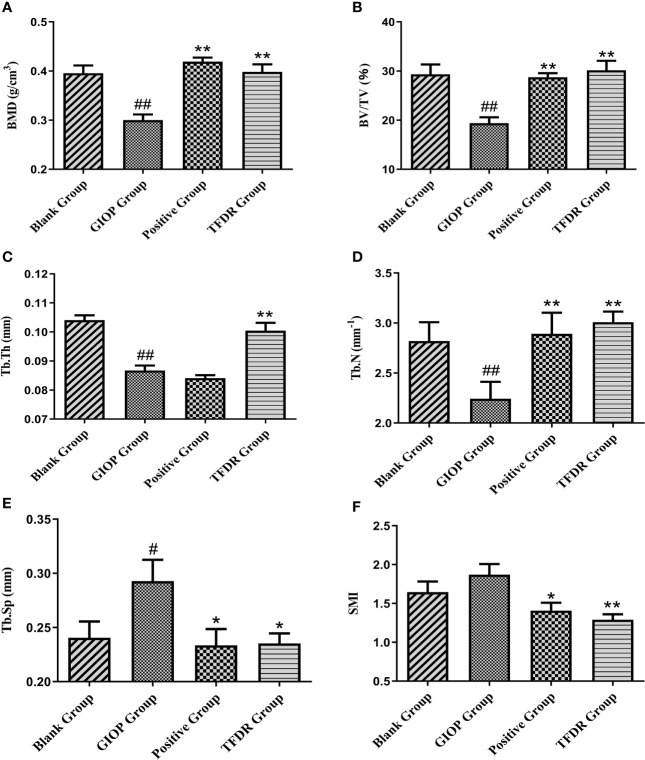
The changes of bone parameters including bone mineral density (BMD) **(A)**, bone value/total value (BV/TV) **(B)**, trabecular thickness (Tb.Th) **(C)**, trabecular number (Tb.N) **(D)**, trabecular spacing (Tb.Sp) **(E)** and structural model index (SMI) **(F)**. Data are expressed as mean ± SEM. *p < 0.05 and **p < 0.01 vs. GIOP group rats; ^#^p < 0.05 and ^##^p < 0.01 vs. blank group rats (n = 6).

Moreover, as indicated by the results of micro-CT scanning (**Figure 9**, **Figure 10B–F**), an improvement in bone structure was observed in the TFDR group compared to the GIOP group and the change (p < 0.05) in some related parameters such as the bone value/total value (BV/TV), trabecular thickness (Tb.Th), trabecular number (Tb.N), trabecular spacing (Tb.Sp), and structural model index (SMI), further confirming the efficacy of TFDR against GIOP.

**Figure 11:**
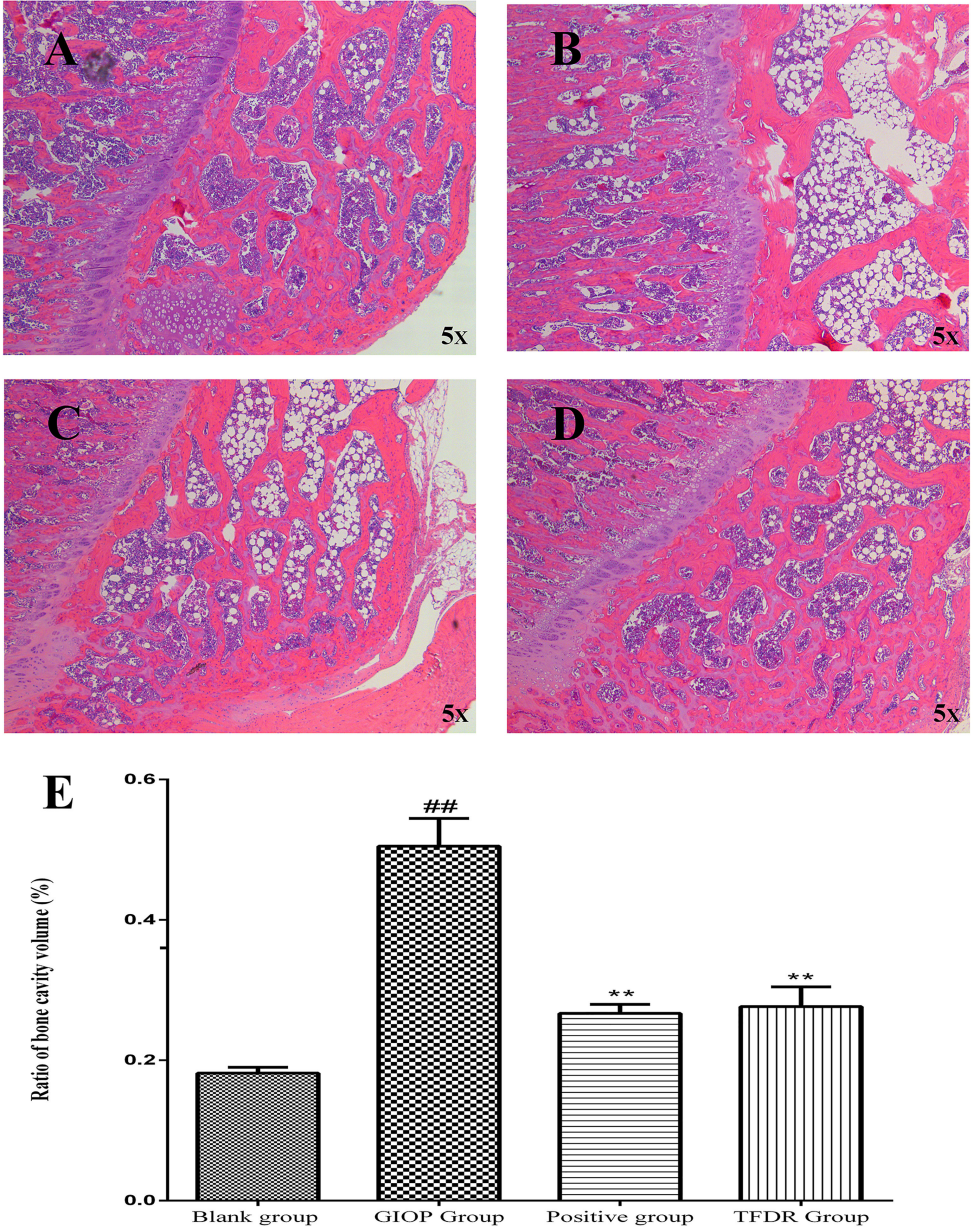
Representative H&E staining of femoral sections from Blank **(A)**, GIOP **(B)**, Positive **(C)** and TFDR **(D)** group. **(E)** Ratio of bone cavity volume regarding representative H&E staining of femoral sections from different groups. Data are expressed as mean ± SEM. ^##^p < 0.01 vs. blank group rats; **p < 0.01 vs. GIOP group rats (n = 4).

HE staining was performed to detect the histological changes in the femur. As shown in **Figure 11**, the femurs in the GIOP group showed an increased number of empty bone lacunae and disordered trabecular structures, whereas those in the TFDR group exhibited complete trabeculae and regular arrangement. These results indicated that TFDR treatment could markedly reverse GC-induced bone structure destruction.

#### 3.3.4 Effects of TFDR on Gene Expression Levels: Validation of Network Pharmacology Prediction

**Figure 12:**
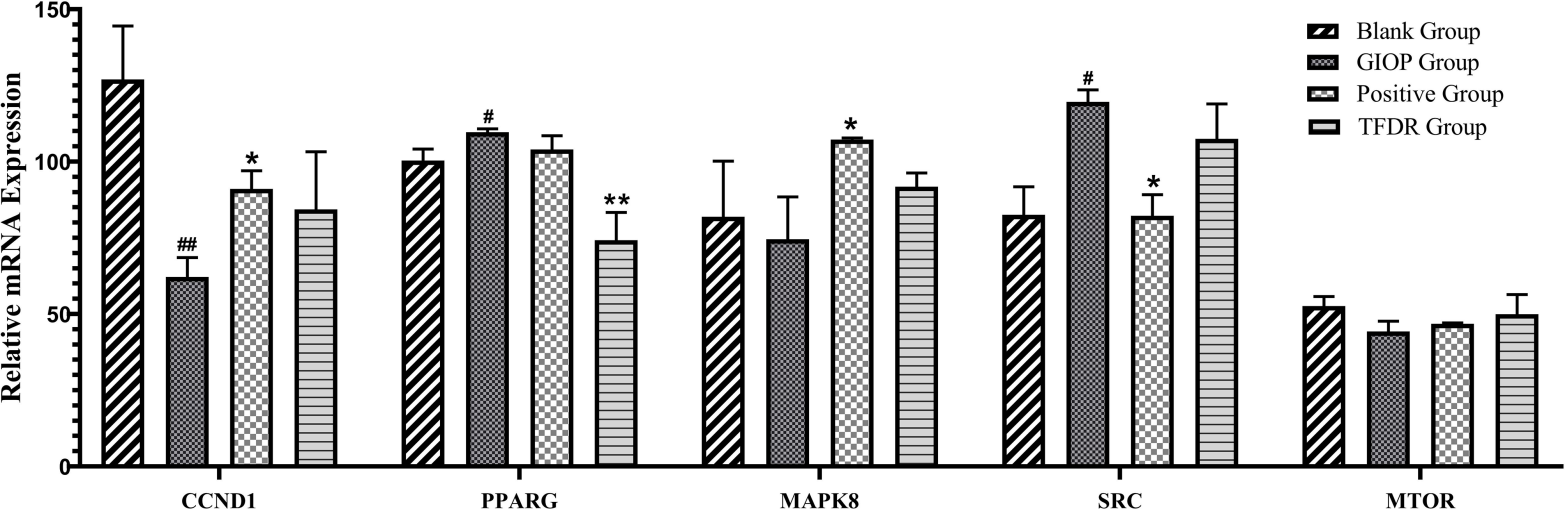
The mRNA expression levels of 6 core targets. Data are expressed as mean ± SEM. *p < 0.05 and **p < 0.01 vs. GIOP group rats; ^#^p < 0.05 and ^##^p < 0.01 vs. blank group rats.

According to the results of network pharmacology prediction, a total of 5 core targets with high degree value were selected for validating by the PCR analyses including CCND1, PPARγ, MAPK8, SRC, MTOR. Compared with GIOP group, the mRNA expression levlels of PPARγ had significant differences in the treatment group (**Figure 12**), which suggested that it might be the potential therapeutic target for the treatment of TFDR against GIOP.

## 4 Discussion

TFDR, which has been widely used in the clinical treatment of orthopedic diseases, is a traditional Chinese medicine with high pharmacological potency (8). However, previous studies mostly focused on the pharmacological effects of TFDR instead of identifying the chemical compounds found in TFDR (27). Thus, to determine its chemical constituents, qualitative analysis by UHPLC-ESI-Q-TOF-MS was performed. A total of 191 ingredients were finally identified, of which naringin and naringenin have been previously regarded as the main bioactive compounds of TFDR (5). Moreover, other potentially bioactive compounds that can be found in blood circulation were identified. Our results indicated a total of 48 compounds including luteolin-7-glucuronide, naringenin, naringenin chalcone, etc. may exert their effect after ingestion and reaching the target sites *via* blood circulation. Besides, according to the results of network pharmacology analysis and qRT-PCR, PPARγ has a close relationship with quercetin, luteolin, isorhamnetin, dehydrodiisoeugenol, nobiletin and kaempferol. Integrating the results of qualitative analysis, we argue that quercetin, luteolin and kaempferol are the potential pharmacodynamic substances of TFDR against GIOP, which deserve to be paid full attention for further analysis.

It has been widely reported that TFDR is safe and effective against POP. Both postmenopausal osteoporosis (POP) and glucocorticoid-induced osteoporosis (GIOP) belong to osteoporosis with an imbalance of bone resorption over bone formation, resulting in reduction of bone mineral density and disruption of bone micro-architecture. Both GIOP and POP are associated with a reduction in bone formation at the cellular level, this effect being quantitatively greater in GIOP (28). However, the predisposing factors of them differ from each other. POP, one of the most common primary osteoporosis, is caused by the decline of estrogen levels in postmenopausal women (29). In contrast to POP, GIOP is a kind of secondary osteoporosis which is mainly caused by using long-term or high-dose glucocorticoids (GCs) in a substantial proportion of patients (4). Currently, it has been widely reported that TFDR therapeutic effects against POP, while if TFDR can treat GIOP have not been fully elucidated. In this research, we studied TFDR-mediated treatment in GIOP rats and its underlying mechanisms. We found that TFDR improved bone mineral density and bone micro-structure as well as changed the levels of bone resorption and bone formation markers in the serum, which in turn improved GIOP. These results favor the extended application of clinical indications of TFDR, laying a solid a foundation for the treatment and prevention of GIOP.

To further explore the exact mechanism of action of TFDR as an anti-osteoporosis agent in rats, network pharmacology analysis, which was based on the analysis of network models and systems biology, was performed to predict the potential targets (30). After integrating the results from the TCMSP database, relevant literature, and qualitative analysis, 13 ingredients were selected for follow-up target screening. Thereafter, by using multiple platforms, GIOP-related targets and TFDR-related targets were collated and highly relevant treatment targets were selected based on the intersections seen in the Venn diagram.

Integrating the results of network pharmacology analysis and literature reports, a total of 5 core related targets including CCND1, PPARγ, MAPK8, SRC, MTOR were finally selected among all the 67 targets obtained. These five protein targets showed a high correlation, which might play an important role in the action of TFDR against GIOP. The prediction results were then preliminarily validated by the qRT-PCR analysis, PPARγ had a highly significant difference in the treatment group.

According to previous studies, the peroxisome proliferator-activated receptor γ gene (PPARγ) was a key regulator of glucose and lipid metabolism as well as can regulate energy balance of the whole body and nutrient sensitivity (31). Because it effected many protein targets related to inflammation and insulin sensitivity, PPARγ has been regarded as major antidiabetic drug target (32). Recently, the tight link between energy metabolism and bone mass has been proved by increasing number of studies (33, 34), PPARγ, as a key target regulating the metabolic network, was also been focused on. Studies have revealed that the mouse with osteocyte-specific PPARγ deletion showed an increased bone mass and a reduced bone marrow adiposity, which indicated PPARγ has a negative effect on bone quality (35). Our results of the qRT-PCR analysis can also give compelling evidences: the mRNA expression level of PPARγ was significantly increased in the GIOP group vs the blank group, while after treating with TFDR, the level showed a significant decline. Our study provided preliminary validation about the influence of TFDR to the mRNA expression level of transcription factor PPARγ, which indicated PPARγ might be a potential therapeutic target underlying TFDR-mediated GIOP treatment while further studies are required to elucidate the detailed mechanism. Furthermore, it has been reported that SRC can directly affect transcription of PPARγ (36, 37), SRC expression increased in TFDR group indirectly confirmed the importance of PPARγ in GIOP.

Besides, the expression levels of CCND1, MAPK8, MTOR in the treatment group showed different degrees of improvement. CCND1 has a relationship with various cancers by regulating cell proliferation and differentiation (38–40). CCND1 expression in the GIOP group significantly decreased and negatively regulated in the positive and TFDR group. MAPK8 was an important target contributing to the pathophysiology of type 2 diabetes (T2D), which can suppress production of insulin and leads to cell apoptosis (41, 42). MTOR played a critical role in cell growth, autophagy and apoptosis. It was also reported to induce osteogenic differentiation to decrease bone loss (5, 43).

The potential mechanisms of TFDR against GIOP were predicted *via* GO and KEGG analyses. It is reasonable that the more targets enriched in one pathway, the more important this pathway was in the treatment of TFDR against GIOP. As the results indicated, pathways in cancer, PI3K-AKT signal pathway and endocrine resist showed a high correlation with TFDR against GIOP. PI3K-AKT pathway plays an important role in regulating cells adhesion, proliferation and apoptosis, engaging in a series of cellular physiological processes. On the one hand, the activation of PI3K-AKT pathway can increase the expression of many signaling molecules associated with bone formation including ALP and BMP-2, which in turn promote the proliferation and differentiation of osteoblasts. On the other hand, its downstream signaling molecules such as RANK and c-FMS can take part in the process of osteoclast-mediated bone resorption (44–49).

Moreover, many studies have investigated that PI3K-AKT signal pathway was a crucial regulator associated with autophagy as well as glucose and lipid metabolism in the treatment of GIOP (43, 48, 50). So, it is not difficult to understand our further study will focus on this pathway in TFDR against GIOP. What we have to admit is that it is hard to validate any specific signaling pathway without a large number of experiments, but we argue that the results of GO/KEGG analyses can provide references to some extent for mining the potential mechanism in further study.

Of note, we incidentally discovered the outstanding lipid-lowering effect of TFDR. To explain this phenomenon, we accessed many related researches and speculated that there was a closely association between bone metabolism and lipid metabolism, which also has been validated by many studies. Fatty acids, cholesterol, phospholipids and several endogenous metabolites (i.e., prostaglandins, oxysterols) have been reported to act on bone cell survival and functions, the bone mineralization process, and critical signaling pathways (51). Besides, long-chain polyunsaturated fatty acids (LCPUFAs) and their metabolites were considered essential factors to support bone and joint health (52). There are produced evidences indicating that lipid plays a crucial role in regulation of bone metabolism (53–57). There was no doubt in our experiment that TFDR could increase BMD of GIOP rats, while further research regarding the specific mechanism and relationship between lipid metabolism and TFDR-induced GIOP treatment were still needed. In the present study, we just wanted to propose a possible assumption about bone metabolism and lipid metabolism based on the fact that TFDR could significantly decrease TC and TG as well as increase BMD in the GIOP rats, which still waited to be further validated in future.

In summary, a total of 191 ingredients were identified *in vitro* and 48 *in vivo*, among them luteolin-7-glucuronide, naringenin, naringenin chalcone, and eriodictyol might be the potential bio-active components in the treatment of TFDR against GIOP. TFDR can significantly improve GCs-induced bone loss and destruction of bone microstructure and it may exert the pharmacodynamic effect by targeting PPARγ, a core target associated with lipid metabolism. As a key regulator of glucose and lipid metabolismas gene PPARγ should be focused on for its close links with the occurrence of osteoporosis, which may also be the possible mechanism underlying TFDR-mediated GIOP treatment.

## Data Availability Statement

The original contributions presented in the study are included in the article/supplementary files, further inquiries can be directed to the corresponding authors.

## Ethics Statement

The animal study was reviewed and approved by the Experimental Animal Center of IMPLAD.

## Author Contributions

FZ and QL designed and performed the experiments. FZ and JW wrote the manuscript. HR, CS, JZ, and QS engaged in carrying out the experiments. XW, YS, and LZ revised the manuscript. All authors contributed to the article and approved the submitted version.

## Funding

This research was supported by Innovation Team and Talents Cultivation Program of National Administration of Traditional Chinese Medicine (ZYYCXTD-C-202003), Science and Technology Innovation Project of China Academy of Chinese Medical Sciences (CI2021A04901), The Fundamental Research Funds for the Central public welfare research institutes (ZZ13-YQ-036) and (ZZ13-YQ-039), and China Postdoctoral Science Foundation (2019M662284).

## Conflict of Interest

The authors declare that the research was conducted in the absence of any commercial or financial relationships that could be construed as a potential conflict of interest.

## Publisher’s Note

All claims expressed in this article are solely those of the authors and do not necessarily represent those of their affiliated organizations, or those of the publisher, the editors and the reviewers. Any product that may be evaluated in this article, or claim that may be made by its manufacturer, is not guaranteed or endorsed by the publisher.
