# Supplementary material for: Total Flavonoids of Drynariae Rhizoma Improve Glucocorticoid-Induced Osteoporosis of Rats: UHPLC-MS-Based Qualitative Analysis, Network Pharmacology Strategy and Pharmacodynamic Validation
Source: Front Endocrinol (Lausanne). 2022 Jun 30;13:920931. doi: 10.3389/fendo.2022.920931 (PMC9279576; doi:10.3389/fendo.2022.920931)
Supplement: Supplementary file 1 [file DataSheet_1.pdf]

Supplementary Table S1. The identification of 191 chemical constituents in TFDR

Peak number	t_R(min)	Identity	Molecular formula	Molecular mass	Error (ppm)	Ion mode
1	1.590	Betaine	$C_5H_{11}NO_2$	117.07893	-0.43	+
2	1.622	Quinic acid	$C_7H_{12}O_6$	192.06328	-0.56	+/-
3	1.624	Guanine	$C_5H_5N_5O$	151.04935	-0.38	+
4	1.627	Sucrose	$C_{12}H_{22}O_{11}$	342.11595	-0.78	+/-
5	1.641	Shikimic acid	$C_7H_{10}O_5$	174.05249	-1.93	-
6	1.779	Hordenine	$C_{10}H_{15}NO$	165.11542	0.33	+
7	2.219	Fumaric acid	$C_4H_4O_4$	116.01078	-1.55	-
8	2.584	Manninotriose	$C_{18}H_{32}O_{16}$	504.16888	-0.31	-
9	2.896	Adenine	$C_5H_5N_5$	135.05435	-1.07	-
10	3.278	Nicotinamide	$C_6H_6N_2O$	122.04796	-0.43	+
11	17.190	Protocatechuic acid	$C_7H_6O_4$	154.02647	-0.89	-
12	17.474	5-Hydroxymethylfurfural	$C_6H_6O_3$	126.03168	-0.12	+
13	17.591	3,4-Dihydroxyphenylethanol	$C_8H_{10}O_3$	154.06291	-0.54	-
14	17.757	Epigallocatechin	$C_{15}H_{14}O_7$	306.07374	-0.68	+/-
15	17.788	Galanthamine hydrobromid	$C_{17}H_{21}NO_3$	287.15209	-0.2	+
16	18.258	Taxifolin 7-rhamnoside	$C_{21}H_{22}O_{11}$	450.11604	-0.38	-
17	18.270	Cryptochlorogenic acid	$C_{16}H_{18}O_9$	354.09487	-0.61	+/-
18	18.278	(R)-Mandelic acid	$C_8H_8O_3$	152.0471	-1.59	-
19	18.279	L-Tryptophan	$C_{11}H_{12}N_2O_2$	204.08975	-0.62	-
20	18.483	Bilobalide	$C_{15}H_{18}O_8$	326.09973	-1.34	-
21	18.511	Cianidanol	$C_{15}H_{14}O_6$	290.07881	-0.77	+
22	18.604	Harmol	$C_{12}H_{10}N_2O$	198.07927	-0.21	+
23	18.612	Procyanidin B_1	$C_{30}H_{26}O_{12}$	578.14203	-0.69	+/-
24	18.663	Protocatechualdehyde	$C_7H_6O_3$	138.03148	-1.55	+/-
25	18.719	Procyanidin B_2	$C_{30}H_{26}O_{12}$	578.14205	-0.66	+/-
26	18.790	Bengenin	$C_{14}H_{16}O_9$	328.07907	-1.09	+/-
27	18.819	Oxyresveratrol	$C_{14}H_{12}O_4$	244.07356	0.02	+
28	18.845	(-)-Gallocatechin	$C_{15}H_{14}O_7$	306.07375	-0.66	+/-
29	18.920	Trigonelline HCl	$C_7H_7NO_2$	137.04765	-0.2	+/-
30	19.324	Esculin	$C_{15}H_{16}O_9$	340.07921	-0.65	+
31	19.354	Epicatechin	$C_{15}H_{14}O_6$	290.07874	-1.01	+/-

32	19.361	Pyrogallol	$C_6H_6O_3$	126.03121	-3.81	-
33	19.371	Chlorogenic acid	$C_{16}H_{18}O_9$	354.0949	-0.52	+
34	19.391	Benzoic acid	$C_7H_6O_2$	122.03668	-0.82	+/-
35	19.423	2''-O-β-L-Galactopyranosylorientin	$C_{27}H_{30}O_{16}$	610.15292	-0.75	+/-
36	19.490	Perillartine	$C_{10}H_{15}NO$	165.11542	0.33	+
37	19.576	4-Methoxyphenylacetic acid	$C_9H_{10}O_3$	166.06277	-1.33	-
38	19.700	Rutin	$C_{27}H_{30}O_{16}$	610.15288	-0.82	+/-
39	19.808	Camphor	$C_{10}H_{16}O$	152.12013	0.07	+
40	19.894	Geniposide	$C_{17}H_{24}O_{10}$	388.13673	-0.55	-
41	19.980	Esculetin	$C_9H_6O_4$	178.02638	-1.28	-
42	20.017	Homoveratrumic acid	$C_{10}H_{12}O_4$	196.07339	-0.84	+/-
43	20.053	Caffeic acid	$C_9H_8O_4$	180.04215	-0.59	+/-
44	20.241	Pinoresinol diglucoside	$C_{32}H_{42}O_{16}$	682.24722	-0.1	-
45	20.302	Pimpinellin	$C_{13}H_{10}O_5$	246.0526	-0.91	+
46	20.335	Noreugenin	$C_{10}H_8O_4$	192.04226	0	+/-
47	20.346	Kaempferol-7-O-β-D-glucopyranoside	$C_{21}H_{20}O_{11}$	448.10036	-0.45	+
48	20.347	Grosvenorine	$C_{33}H_{40}O_{19}$	740.21623	-0.2	+
49	20.350	Kaempferol-3-O-rutinoside	$C_{27}H_{30}O_{15}$	594.15816	-0.52	+/-
50	20.385	(+)-Magnoflorine	$C_{20}H_{23}NO_4$	341.16252	-0.54	+
51	20.397	p-Hydroxybenzaldehyde	$C_7H_6O_2$	122.03662	-1.31	+/-
52	20.437	Camelliaside B	$C_{32}H_{38}O_{19}$	726.20064	-0.12	+/-
53	20.460	Coumarin	$C_9H_6O_2$	146.03674	-0.26	+
54	20.471	Taxifolin	$C_{15}H_{12}O_7$	304.05812	-0.59	+
55	20.514	Phloridzin	$C_{21}H_{24}O_{10}$	436.13685	-0.22	+/-
56	20.675	Germacrone	$C_{15}H_{22}O$	218.16694	-0.56	+
57	20.681	Rhaponticin	$C_{21}H_{24}O_9$	420.14134	-1.66	-
58	20.697	7,8-Dihydroxycoumarin	$C_9H_6O_4$	178.02638	-1.29	-
59	20.701	7-Methoxycoumarin	$C_{10}H_8O_3$	176.0474	0.29	+
60	20.718	5,7-Dihydroxy-4-methylcoumarin	$C_{10}H_8O_4$	192.04222	-0.22	+/-
61	20.784	Orsellinic acid	$C_8H_8O_4$	168.04217	-0.53	+/-
62	20.871	7-Methoxy-4-methylcoumarin	$C_{11}H_{10}O_3$	190.06297	-0.1	+
63	20.943	Leucoside	$C_{26}H_{28}O_{15}$	580.14258	-0.41	+/-
64	21.022	Orientin	$C_{21}H_{20}O_{11}$	448.10021	-0.78	-
65	21.064	Cinnamic acid	$C_9H_8O_2$	148.05249	0.42	+

66	21.188	Hesperetin	$C_{16}H_{14}O_6$	302.079	-0.14	+
67	21.371	Procyanidin A_2	$C_{30}H_{24}O_{12}$	576.12661	-0.28	+/-
68	21.440	Gentiopicrin	$C_{16}H_{20}O_9$	356.11022	-1.44	-
69	21.507	Vanillin	$C_8H_8O_3$	152.04727	-0.47	+/-
70	21.516	Dihydroartemisinin	$C_{15}H_{24}O_5$	284.16185	-1.85	-
71	21.544	Safflomin A	$C_{27}H_{32}O_{16}$	612.16859	-0.72	-
72	21.600	p-Coumaric acid	$C_9H_8O_3$	164.0472	-0.87	+/-
73	21.662	Engeletin	$C_{21}H_{22}O_{10}$	434.12093	-0.85	+
74	21.663	Eriodictyol	$C_{15}H_{12}O_6$	288.06304	-1.22	+/-
75	21.665	Eriocitrin	$C_{27}H_{32}O_{15}$	596.17339	-1.23	+/-
76	21.665	Troxerutin	$C_{33}H_{42}O_{19}$	742.23132	-0.96	+
77	21.679	Artemisinin	$C_{15}H_{22}O_5$	282.14653	-0.7	+
78	21.695	o-Veratraldehyde	$C_9H_{10}O_3$	166.06292	-0.46	+
79	21.718	Astilbin	$C_{21}H_{22}O_{11}$	450.11574	-1.05	+/-
80	21.833	4-Methyl-6,7-dihydroxycoumarin	$C_{10}H_8O_4$	192.04201	-1.32	-
81	21.849	Lonicerin	$C_{27}H_{30}O_{15}$	594.15829	-0.3	+/-
82	21.917	Cinnamaldehyde	C_9H_8O	132.05745	-0.47	+
83	21.941	3,5-Dimethoxy-4-hydroxybenzaldehyde	$C_9H_{10}O_4$	182.05794	0.17	+
84	21.956	Tetrahydroxyxanthone	$C_{13}H_8O_6$	260.03179	-1.15	+/-
85	21.959	Astringin	$C_{20}H_{22}O_9$	406.12612	-0.64	+/-
86	21.983	Isoquercitrin	$C_{21}H_{20}O_{12}$	464.09546	-0.03	+/-
87	21.995	Kaempferitrin	$C_{27}H_{30}O_{14}$	578.16354	-0.02	+/-
88	22.021	Cynaroside	$C_{21}H_{20}O_{11}$	448.10012	-0.98	+/-
89	22.049	Scutellarin	$C_{21}H_{18}O_{12}$	462.08034	1.1	-
90	22.215	Pogostone	$C_{12}H_{16}O_4$	224.1048	-0.27	+
91	22.236	Salidroside	$C_{14}H_{20}O_7$	300.1205	-1.35	-
92	22.354	Isochlorogenic acid B	$C_{25}H_{24}O_{12}$	516.12636	-0.81	-
93	22.421	(+)-Pinoresinol 4-O-glucoside	$C_{26}H_{32}O_{11}$	520.19353	-1.79	-
94	22.461	Xanthoxyline	$C_{10}H_{12}O_4$	196.07338	-0.91	+
95	22.581	Isoeugenol acetate	$C_{12}H_{14}O_3$	206.09421	-0.43	+
96	22.630	Naringenin chalcone	$C_{15}H_{12}O_5$	272.06804	-1.59	+
97	22.631	Naringin	$C_{27}H_{32}O_{14}$	580.17834	-1.49	+/-
98	22.641	Atractylenolide II	$C_{15}H_{20}O_2$	232.14605	-1.2	+
99	22.682	Sophoricoside	$C_{21}H_{20}O_{10}$	432.10546	-0.44	+

100	22.687	Rhoifolin	$C_{27}H_{30}O_{14}$	578.16332	-0.41	+/-
101	22.691	Ligustroflavone	$C_{33}H_{40}O_{18}$	724.22099	-0.65	+
102	22.751	Astragalin	$C_{21}H_{20}O_{11}$	448.1001	-1.02	+/-
103	22.994	Apigenin-7-O-β-D-glucoside	$C_{21}H_{20}O_{10}$	432.10509	-1.3	-
104	23.006	Isoalantolactone	$C_{15}H_{20}O_2$	232.14606	-1.15	+
105	23.074	Linderalactone	$C_{15}H_{16}O_3$	244.10982	-0.5	+
106	23.110	Nardosinone	$C_{15}H_{22}O_3$	250.15663	-1.04	+
107	23.115	Isochlorogenic acid C	$C_{25}H_{24}O_{12}$	516.12623	-1.05	-
108	23.118	Piceatannol	$C_{14}H_{12}O_4$	244.07304	-2.12	-
109	23.123	Sauchinone	$C_{20}H_{20}O_6$	356.12578	-0.58	+
110	23.124	5,7-Dihydroxychromone	$C_9H_6O_4$	178.02645	-0.91	+/-
111	23.149	Neohesperidin	$C_{28}H_{34}O_{15}$	610.18928	-0.8	-
112	23.167	Poncirin	$C_{28}H_{34}O_{14}$	594.19442	-0.73	+/-
113	23.252	Demethoxycurcumin	$C_{20}H_{18}O_5$	338.11526	-0.48	+
114	23.363	Sinapic acid	$C_{11}H_{12}O_5$	224.06836	-0.51	+/-
115	23.378	Azelaic acid	$C_9H_{16}O_4$	188.10469	-0.88	+/-
116	23.39	Isofraxidin	$C_{11}H_{10}O_5$	222.05255	-1.22	+
117	23.421	β-Asarone	$C_{12}H_{16}O_3$	208.10983	-0.53	+/-
118	23.707	5-Hydroxy-1-tetralone	$C_{10}H_{10}O_2$	162.06794	-0.85	+/-
119	23.788	Isosakuranetin	$C_{16}H_{14}O_5$	286.08299	-3.95	+
120	23.834	Kaempferol	$C_{15}H_{10}O_6$	286.04747	-0.93	+
121	23.910	Diffractic acid	$C_{20}H_{22}O_7$	374.13614	-1.1	+/-
122	23.971	Ethyl 3,4-dihydroxybenzoate	$C_9H_{10}O_4$	182.05777	-0.77	-
123	24.079	Dehydroandrographolide	$C_{20}H_{28}O_4$	332.19858	-0.53	+
124	24.169	Ethyl ferulate	$C_{12}H_{14}O_4$	222.08899	-1	-
125	24.488	Curcumol	$C_{15}H_{24}O_2$	236.17745	-0.78	+
126	24.584	Ligustilide	$C_{12}H_{14}O_2$	190.09924	-0.71	+
127	24.746	Rosmarinic acid	$C_{18}H_{16}O_8$	360.0843	-0.6	-
128	24.756	Linderane	$C_{15}H_{16}O_4$	260.10463	-0.87	+
129	24.973	Atractylenolide I	$C_{15}H_{18}O_2$	230.13056	-0.54	+
130	24.985	Artemisinic acid	$C_{15}H_{22}O_2$	234.16182	-0.66	+
131	25.042	Anisic aldehyde	$C_8H_8O_2$	136.05245	0.12	+
132	25.176	Pinocembrin	$C_{15}H_{12}O_4$	256.07338	-0.69	+
133	25.178	Abscisic acid	$C_{15}H_{20}O_4$	264.13592	-0.89	+/-
134	25.203	Gracillin	$C_{45}H_{72}O_{17}$	884.47667	-0.32	+

135	25.706	Emodin-8-O-β-D-glucopyranoside	$C_{21}H_{20}O_{10}$	432.10509	-1.3	-
136	25.736	Hecogenin	$C_{27}H_{42}O_{4}$	430.30822	-0.2	+
137	25.739	Luteolin	$C_{15}H_{10}O_{6}$	286.04742	-1.1	+/-
138	25.796	Quercetin	$C_{15}H_{10}O_{7}$	302.04248	-0.57	+/-
139	26.316	Ethyl caffeate	$C_{11}H_{12}O_{4}$	208.07329	-1.31	-
140	26.883	3-Butylidenephthalide	$C_{12}H_{12}O_{2}$	188.08367	-0.31	+
141	27.194	Perillene	$C_{10}H_{14}O$	150.10447	0.04	+
142	27.455	Naringenin	$C_{15}H_{12}O_{5}$	272.06804	-1.6	+/-
143	27.650	Apigenin	$C_{15}H_{10}O_{5}$	270.05266	-0.59	+/-
144	27.734	Farrerol	$C_{17}H_{16}O_{5}$	300.09953	-0.8	+
145	27.880	Curcumenol	$C_{15}H_{22}O_{2}$	234.16183	-0.66	+
146	28.198	Isorhamnetin	$C_{16}H_{12}O_{7}$	316.05814	-0.5	+/-
147	28.334	Amentoflavone	$C_{30}H_{18}O_{10}$	538.08955	-0.83	-
148	28.441	Arglabin	$C_{15}H_{18}O_{3}$	246.12543	-0.67	+
149	28.540	2-Adamantanone	$C_{10}H_{14}O$	150.10446	-0.02	+
150	28.592	Parthenolide	$C_{15}H_{20}O_{3}$	248.14092	-1.32	+
151	29.108	5,7,3'-Trihydroxy-6,4',5'-trimethoxyflavone	$C_{18}H_{16}O_{8}$	360.08455	0.1	+/-
152	29.400	Demethylwedelolactone	$C_{15}H_{8}O_{7}$	300.02666	-1.16	-
153	29.458	Dehydrocostus lactone	$C_{15}H_{18}O_{2}$	230.13056	-0.54	+
154	30.011	Micheliolide	$C_{15}H_{20}O_{3}$	248.14092	-1.32	+
155	30.427	Clareolide	$C_{16}H_{26}O_{2}$	250.1931	-0.71	+
156	31.507	Isosteviol	$C_{20}H_{30}O_{3}$	318.21922	-0.85	+
157	32.459	Nobiletin	$C_{21}H_{22}O_{8}$	402.13121	-0.63	+
158	32.468	6-Shogaol	$C_{17}H_{24}O_{3}$	276.17244	-0.37	+
159	32.824	Arenobufagin	$C_{24}H_{32}O_{6}$	416.21942	-1.13	+/-
160	33.149	6-Gingerol	$C_{17}H_{26}O_{4}$	294.18287	-0.8	-
161	33.694	Neoandrographolide	$C_{26}H_{40}O_{8}$	480.27201	-0.64	-
162	34.329	Tangeretin	$C_{20}H_{20}O_{7}$	372.12062	-0.75	+
163	34.971	Atractylodin	$C_{13}H_{10}O$	182.07309	-0.4	+
164	35.263	Lindenenol	$C_{15}H_{18}O_{2}$	230.13056	-0.54	+
165	36.199	Emodin	$C_{15}H_{10}O_{5}$	270.05235	-1.75	-
166	36.219	Spiculisporic acid	$C_{17}H_{28}O_{6}$	328.18822	-1.14	-
167	36.663	Deoxyandrographolide	$C_{20}H_{30}O_{4}$	334.21422	-0.57	+
168	36.744	Dihydrotanshinone I	$C_{18}H_{14}O_{3}$	278.09407	-0.8	+

169	39.464	Senkyunolide A	$C_{12}H_{16}O_2$	192.11496	-0.35	+
170	40.304	Cryptotanshinone	$C_{19}H_{20}O_3$	296.1411	-0.48	+
171	40.387	α-Linolenic acid	$C_{18}H_{30}O_2$	278.22438	-0.71	+
172	41.782	Cafestol	$C_{20}H_{28}O_3$	316.20119	-8.4	+
173	41.873	Abietic Acid	$C_{20}H_{30}O_2$	302.22423	-1.16	+
174	43.744	α-Cyperone	$C_{15}H_{22}O$	218.1669	-0.75	+
175	44.046	Tanshinone IIA	$C_{19}H_{18}O_3$	294.12539	-0.71	+
176	44.050	(+)-Usniacin	$C_{18}H_{16}O_7$	344.0892	-1.18	+/-
177	46.200	Rubescensin A	$C_{20}H_{28}O_6$	364.18596	-7.22	+
178	46.393	Linolenic acid ethyl ester	$C_{20}H_{34}O_2$	306.25572	-0.51	+
179	46.716	18 β-Glycyrrhetintic Acid	$C_{30}H_{46}O_4$	470.33928	-0.7	+/-
180	47.619	Dehydrotumulosic acid	$C_{31}H_{48}O_4$	484.35527	0.03	+
181	48.473	Panaxtriol	$C_{30}H_{52}O_4$	476.38622	-0.72	+
182	48.864	Alisol B 23-acetate	$C_{32}H_{50}O_5$	514.36327	-4.97	+
183	49.113	Ginkgolic Acid C15:1	$C_{22}H_{34}O_3$	346.25014	-1.9	-
184	50.403	Roburic acid	$C_{30}H_{48}O_2$	440.36516	-0.61	+
185	50.404	α-Boswellic acid	$C_{30}H_{48}O_3$	456.36003	-0.68	+
186	50.800	Wilforlide A	$C_{30}H_{46}O_3$	454.34437	-0.72	+
187	50.955	Ginkgolic acid C17-1	$C_{24}H_{38}O_3$	374.28197	-0.35	-
188	52.356	Gitogenin	$C_{27}H_{44}O_4$	432.3235	-1.06	+
189	53.078	20(R)-Protopanaxdiol	$C_{30}H_{52}O_3$	460.39202	0.8	+
190	62.942	Lupenone	$C_{30}H_{48}O$	424.3701	-0.97	+
191	63.505	Melamine	$C_3H_6N_6$	126.06533	-0.49	+

Supplementary Table S2. The identification of 48 chemical constituents ingested into blood

Peak number	t_R(min)	Identity	Molecular formula	Molecular mass	Error (ppm)	Ion mode
1	1.66	L-Glutamic acid	$C_5H_9NO_4$	147.05292	-1.65	+/-
2	1.686	Maleic acid	$C_4H_4O_4$	116.01063	-2.81	-
3	5.726	Uridine	$C_9H_{12}N_2O_6$	244.06904	-2.02	-
4	18.187	3,4-Dihydroxyphenylethanol	$C_8H_{10}O_3$	154.06267	-2.13	-
5	18.201	L-Tryptophan	$C_{11}H_{12}N_2O_2$	204.08954	-1.67	+/-
6	19.84	Protocatechuic acid	$C_7H_6O_4$	154.02638	-1.48	-
7	21.613	p-Coumaric acid	$C_9H_8O_3$	164.04706	-1.75	+/-
8	22.794	Scutellarin	$C_{21}H_{18}O_{12}$	462.07925	-1.24	+/-
9	23.039	Luteolin 7-glucuronide	$C_{21}H_{18}O_{12}$	462.07924	-1.27	+/-
10	23.321	Quercetin 7-rhamnoside	$C_{21}H_{20}O_{11}$	448.10002	-1.21	-
11	23.756	4-Hydroxybenzoic acid	$C_7H_6O_3$	138.03146	-1.68	-
12	24.806	Tetrahydroxyxanthone	$C_{13}H_8O_6$	260.03428	8.45	-
13	25.017	Daidzein	$C_{15}H_{10}O_4$	254.05755	-1.39	-
14	25.026	Herbacetin	$C_{15}H_{10}O_7$	302.04466	6.65	-
15	25.902	Ganoderic acid G	$C_{30}H_{44}O_8$	532.30647	5.36	-
16	27.463	Naringenin chalcone	$C_{15}H_{12}O_5$	272.06807	-1.49	+/-
17	27.64	Genistein	$C_{15}H_{10}O_5$	270.05256	-0.96	+/-
18	31.818	Andrographolide	$C_{20}H_{30}O_5$	350.20894	-1.11	-
19	34.755	Deoxycholic acid	$C_{24}H_{40}O_4$	392.29216	-1.28	-
20	3.884	Cytosine	$C_4H_5N_3O$	111.04316	-0.9	+
21	12.533	L-Phenylalanine	$C_9H_{11}NO_2$	165.07889	-0.54	+
22	19.438	6-Hydroxyindole	C_8H_7NO	133.05268	-0.62	+
23	19.447	3-Furfuryl 2-pyrrolecarboxylate	$C_{10}H_9NO_3$	191.05807	-0.89	+
24	19.821	L-Abrine	$C_{12}H_{14}N_2O_2$	218.10532	-0.96	+
25	19.898	Eriodictyol	$C_{15}H_{12}O_6$	288.06311	-0.97	+
26	20.945	Anisic aldehyde	$C_8H_8O_2$	136.05232	-0.77	+
27	23.313	Naringenin	$C_{15}H_{12}O_5$	272.06807	-1.48	+
28	23.583	Berberine	$C_{20}H_{17}NO_4$	335.11513	-1.88	+
29	23.753	Emodin-3-methyl ether/Physcion	$C_{16}H_{12}O_5$	284.06822	-0.88	+
30	24.909	5-Hydroxy-1-tetralone	$C_{21}H_{21}NO_4$	351.14668	-1.06	+
31	25.163	Arglabin	$C_{20}H_{17}NO_4$	335.11531	-1.33	+

32	26.732	Isopropyl 4-Hydroxybenzoate	$C_{15}H_{18}O_3$	246.12525	-1.39	+
33	27.465	14-Deoxyandrographolide	$C_{15}H_{12}O_5$	272.06801	-1.7	+
34	27.642	Isoalantolactone	$C_{15}H_{10}O_5$	270.05242	-1.51	+
35	28.877	α-Cyperone	$C_{10}H_{12}O_3$	180.07823	-2.28	+
36	30.286	Artemisinic acid	$C_{20}H_{30}O_4$	334.21358	-2.48	+
37	30.897	Glycitein	$C_{15}H_{20}O_2$	232.14613	-0.88	+
38	31.856	Sarsasapogenin	$C_{15}H_{22}O$	218.16672	-1.59	+
39	31.982	α-Linolenic acid	$C_{15}H_{22}O_2$	234.16173	-1.07	+
40	32.143	Isosteviol	$C_{16}H_{12}O_5$	284.06819	-0.99	+
41	32.903	Clareolide	$C_{27}H_{44}O_3$	416.32854	-1.21	+
42	32.998	Atractylenolide III	$C_{18}H_{30}O_2$	278.22411	-1.7	+
43	33.105	Deoxyandrographolide	$C_{20}H_{30}O_3$	318.21907	-1.33	+
44	34.289	Ginkgolic acid C17-1	$C_{16}H_{26}O_2$	250.19309	-0.76	+
45	35.792	Senkyunolide A	$C_{15}H_{20}O_3$	248.14091	-1.36	+
46	36.242	Dehydroandrographolide	$C_{20}H_{30}O_4$	334.21411	-0.89	+
47	36.856	Ginkgolic acid (C13:0)	$C_{24}H_{38}O_3$	374.28165	-1.18	+
48	37.021	Lupenone	$C_{12}H_{16}O_2$	192.11482	-1.07	+

Supplementary Table S3. The information of 13 compounds in TFDR

MOLID	Compound
MOL001040	(2R)-5,7-dihydroxy-2-(4-hydroxyphenyl)chroman-4-one
MOL001978	Aureusidin
MOL000422	kaempferol
MOL004328	naringenin
MOL005190	eriodictyol
MOL000006	luteolin
MOL009091	xanthogalenol
MOL009070	Luteolin 7-O-glucuronide
MOL009088	Neoeriocitrin
MOL000561	Astragalin
MOL005812	Naringin
MOL000098	Quercetin
MOL009085	(2S)-2-(2,4-dihydroxyphenyl)-7-hydroxy-8-[(2R)-2-isopropenyl-5-methylhex -4-enyl]-5-methoxy-4-chromanone
